# Automated scoring of adult ego development from sentence completions: interpretable maturity indicators derived by evolutionary prompt optimization

**DOI:** 10.3389/fpsyg.2026.1853871

**Published:** 2026-08-27

**Authors:** Tamas Madl, Susanne R. Cook-Greuter

**Affiliations:** 1 Austrian Research Institute for Artificial Intelligence and University of Vienna, Vienna, Austria; 2 Cook-Greuter and Associates, Wayland, MA, United States

**Keywords:** adult development, computational psychometrics, ego development, evolutionary algorithm, large language models, maturity indicators, personality assessment, sentence completion test

## Abstract

**Introduction:**

Ego Development Theory (EDT) describes systematic changes in how adults make meaning, but its principal measure, the Sentence Completion Test (SCT), requires resource-intensive expert scoring.

**Methods:**

We developed an automated scoring approach in which an evolutionary algorithm derives a small set of interpretable maturity indicators, each scored from sentence-completion responses by a domain-fine-tuned language model; a classifier trained on the resulting scores then predicts developmental stage. Performance was evaluated on an expert-scored dataset never used for feature discovery (*N* = 851), in a fully blinded comparison with an independently trained scorer (*N* = 47), and against external criteria (*N* = 446).

**Results:**

The classifier agreed exactly with expert ratings in 86.1% of cases (quadratic-weighted κ = 0.93). By contrast, zero-shot prompting of two frontier language models with the full scoring manual yielded only 40.1–44.9% agreement, indicating that expert-comparable scoring is not achieved by prompting alone. The classifier's agreement replicated at 85.1% in the blinded comparison. In external validation, the indicators correlated with personality, coherence, and autonomy measures in theoretically expected patterns, and explained substantial additional variance in expert-rated developmental stage beyond Big Five traits (Δ*R*^2^ = 0.59). In further analyses, they were also consistent with EDT's predicted alternation of differentiation and integration across stages. The Language Complexity indicator agreed with human-rated Integrative Complexity (ICC = 0.73 and 0.77 in two corpora). Exploratory analyses in five non-SCT corpora suggested the indicators predict domain-relevant behavior in other text genres.

**Discussion:**

These results support automated ego-development scoring at agreement levels comparable to trained raters, and the indicators offer interpretable candidate dimensions of adult development for confirmatory and population-scale research.

## 1 Introduction

Understanding the trajectory of human psychological development across the lifespan is an important theme in personality psychology (e.g., McAdams et al., 2021; Costa et al., 2019; Lanning et al., 2018; Roberts et al., 2006). While various models exist, stage theories of adult development offer compelling frameworks for understanding shifts in complexity, perspective-taking, and meaning-making (Kjellström and Stålne, 2017; Pfaffenberger, 2005). These developmental patterns, particularly toward greater maturity, are important not only for individual wellbeing (Staudinger, 2020) but may be relevant for societal challenges and needs. For instance, developmentally mature leadership, often associated with later stage development, has been linked empirically to enhanced organizational effectiveness (Eigel and Kuhnert, 2005), superior 360° performance ratings (Strang and Kuhnert, 2009), higher success rates in organizational transformations (Torbert and Livne-Tarandach, 2009), and stronger engagement with environmental and social responsibility initiatives (Boiral et al., 2014; Salvetti and Nijhof, 2020; Cardillo, 2023). Such findings underscore the importance of robust methods for assessing adult development.

Among the most influential and empirically grounded stage theories is Ego Development Theory (EDT), initially formulated by Loevinger (1966), Loevinger and Wessler (1970), Loevinger (1976), Hy and Loevinger (1996) and significantly extended by Cook-Greuter (1999), Cook-Greuter (2004). EDT conceptualizes the ego as a core meaning-making system that evolves through a hierarchical sequence of stages. Each subsequent stage represents a more complex and differentiated way of constructing reality, integrating internal states with external experiences, reflecting an increasing capacity for self-awareness and perspective-taking (Cook-Greuter, 1999, 2004; Singleton et al., 2021).

The primary empirical methodology for assessing EDT relies on Sentence Completion Tests (SCTs), semi-projective measures originally developed by Loevinger and Wessler (1970), Loevinger (1985) as the Washington University Sentence Completion Test of Ego Development (WUSCT). Participants complete sentence stems (typically 36). Expert raters score these responses by evaluating their structural complexity, conceptual nuances, and meaning-making patterns against comprehensive scoring manuals and exemplars (Loevinger and Wessler, 1970; Hy and Loevinger, 1996) to assess the underlying ego development stage reflected in the language. Item scores are then aggregated quantitatively across an individual's complete set of 36 completions to determine an overall stage, expressed as a Total Protocol Rating (TPR)—a single number expressing the inferred center of gravity or stage of ego development (Cook-Greuter, 1999; Hy and Loevinger, 1996).

Cook-Greuter (1999) and Cook-Greuter (2004) significantly revised Loevinger's scoring system and theoretical framing, resulting in the Maturity Assessment Profile (MAP) scoring system for the SCT. This revision, initiated in the mid-1980s, was driven by empirical SCT data, particularly at later stages, that could not be adequately explained or scored using the existing theory and manuals (Cook-Greuter, 1999). Key revisions included restructuring the scoring criteria around increasing capacity for perspective-taking on the self as a central organizing principle, reinterpreting later stages as qualitatively distinct modes of meaning-making rather than extensions of autonomy, and adding two post-autonomous stages not described by Loevinger (Cook-Greuter, 1999). The reliability, validity, and applicability of the MAP system have been established across diverse populations through extensive research that was initially grounded in Cook-Greuter's dissertation analyzing 4,500 MAP protocols. It was significantly expanded since through work by Cook-Greuter and certified raters, encompassing thousands more protocols (see e.g., Cook-Greuter, 2004, 2011; Binder, 2023).

The developmental stages used in this study originate in Loevinger's (1976) ego development framework. Cook-Greuter (1999) extended this sequence into the post-autonomous range (stages 5/6 and 6), and provided descriptive stage names emphasizing the developmental capacity at each level (stage number and earlier Cook-Greuter and Loevinger stage names in brackets): Self-Centric (Stage 2/3, “Opportunist,” “Self-Protective”), Group-Centric (Stage 3, “Diplomat,” “Conformist”), Skill-Centric (3/4, “Expert,” “Self-Aware”), Self-Determining (4, “Achiever,” “Conscientious”), Self-Questioning (4/5, “Pluralist,” “Individualistic”), Self-Actualizing (5, “Strategist,” “Autonomous”), Construct-Aware (5/6, “Magician,” “Integrated”), and Unitive (6, “Unitive,” no Loevinger equivalent). We use Cook-Greuter's nomenclature throughout (Cook-Greuter, 2018).

Conceptually within this framework, the ego functions akin to a central processing unit or a “tireless narrator,” responsible for making sense of experience by metabolizing and integrating information from both external reality and the inner world (Cook-Greuter, 1999). The developmental progression can thus be viewed as a hierarchical sequence of nine distinct “self-stories,” each reflecting a more complex and sophisticated capacity for perspective-taking. Subsequent stages generally embody greater flexibility, self-awareness, capability, and discernment, moving from simpler, rule-oriented perspectives toward nuanced, self-aware, and ultimately transpersonal understandings of the self and the world (Cook-Greuter, 1999, 2004).

Despite the established psychometric properties and theoretical richness of the SCT/MAP when scored by experts, the reliance on manual scoring presents significant challenges that limit the widespread application of EDT. The training required to achieve the 85% exact-match agreement necessary for formal rater certification is extensive, time-consuming, and costly. Furthermore, maintaining this reliability necessitates continuous calibration among those raters (Cook-Greuter, 2011). Finally, this intricate, manual analysis restricts processing throughput, creating a severe bottleneck for both research scalability and broader clinical application. These challenges underscore a need for an accessible, automated approach to EDT scoring.

Computational methods, particularly advances in natural language processing and large language models (LLMs), offer potential avenues to address these challenges. However, preliminary work and theoretical considerations suggest that simply providing LLMs with scoring manuals and asking them to score SCT responses often yields insufficient accuracy: in initial multi-model comparisons, Bronlet (2025) observed stage differences of up to three full stages and standard deviations of differences between 0.63 and 0.98 (consistent with our own prompting experiments; see Section 3). The subtlety and depth required to interpret meaning-making structures appear to go beyond pattern matching based on stage descriptions or manual examples alone. This aligns with the well-established claim that projective tests of adult development assess “structure, not content” (Kegan, 1982; Cook-Greuter, 1999). These results highlight the need for more sophisticated computational approaches that can capture the deeper developmental patterns within language.

This paper introduces a data-driven approach to automated SCT scoring using evolutionary algorithms. To our knowledge, the resulting model is the first automated method to reach agreement with expert EDT ratings comparable to that between trained human raters.

Instead of attempting to encode complex scoring rules directly or to mimic human raters, our approach evolves prompts that define candidate dimensions—“maturity indicators”—within the semantic space of responses. These dimensions are not predetermined by theory but emerge from an optimization process that maximizes the separability of expert-rated EDT stages in a large dataset. This method aims to achieve accurate assessment while yielding interpretable candidate dimensions of ego development whose psychological meaning can be tested against external criteria—capturing meaningful variance beyond superficial linguistic features. We emphasize at the outset that these dimensions are validated here as measurement constructs; whether they correspond to the latent structure along which development actually unfolds is a stronger claim that we treat as hypothesis-generating (see Section 4).

To validate this approach and demonstrate its utility, we pursued several aims. First, we developed the evolutionary AI model and evaluated its scoring accuracy against expert human ratings on a benchmark dataset, comparing its performance to established inter-rater reliability benchmarks and to baseline approaches including state-of-the-art frontier language models. Second, we investigated the nature of the discovered maturity indicators, assessing their interpretability and examining how expert-scored responses organize within the AI-derived feature space. Third, we validated the psychological meaningfulness of the discovered indicators against external criteria—personality traits, interpersonal style, indices of personality coherence, and autonomous functioning (measures detailed in Section 2)—assessing both zero-order associations and incremental validity beyond Big Five traits, and testing a theoretical prediction regarding alternating differentiation and integration phases across stages. Fourth, we validated a separate Language Complexity indicator against Integrative Complexity (Suedfeld et al., 1992), a human-coded measure of differentiation and integration in reasoning, using expert-scored corpora, and tested whether the full set of indicators predicts stage beyond linguistic complexity alone. Fifth, we conducted exploratory analyses to assess whether the indicators generalize to non-SCT text genres. Finally, we conducted an independent blinded replication with a separately trained scorer to assess generalization.

The validation draws on seven datasets: a large discovery corpus scored in Loevinger's tradition, an expert-scored evaluation corpus based on Cook-Greuter's system, an independent sample with rich personality and coherence measures, a stream-of-consciousness corpus for testing alternating developmental patterns, two corpora scored for Integrative Complexity, and an independent blinded replication sample.

Throughout, we distinguish three levels of claim that this work supports to different degrees: (i) *measurement automation*—that an AI system can reproduce expert EDT scoring with human-comparable agreement (directly tested here); (ii) *construct validity*—that the discovered indicators track maturity-relevant variance, evidenced by theoretically patterned associations with external criteria never seen during model development; and (iii) *structural discovery*—that these indicators reflect the latent organization of ego development itself. The first two claims are the focus of our empirical program; the third is offered as a hypothesis-generating interpretation whose confirmation requires dedicated designs (see Section 4.1). Section 2 details the methodology, Section 3 presents results, and Section 4 discusses implications.

## 2 Materials and methods

### 2.1 Participants and data

This study utilized archival datasets comprising Sentence Completion Test (SCT) responses or related text data, enabling a multi-stage approach to model development and validation. Our strategy involved: (1) using a large corpus scored according to Loevinger's tradition for feature discovery via our evolutionary algorithm (Vikhar, 2016) approach (Dataset 1); (2) evaluating scoring accuracy against an expert-scored corpus based on Cook-Greuter's system (Dataset 2); (3) assessing construct validity through correlations in a contemporary dataset with SCT and personality/coherence measures (Dataset 3); (4) examining theoretical developmental patterns using AI-scored text from a large stream-of-consciousness dataset (Dataset 4); and (5) conducting an independent blinded replication with separately trained raters (Dataset 7). No new data were collected. Table 1 (Section 2.3) summarizes each dataset's criterion source and role in the pipeline.

**Table 1:** Datasets, criterion sources, and their roles in the modeling pipeline.

Dataset	*N*	Criterion/labels	Role in pipeline	Seen during evolution?
1 Loevinger composite	43,933 items	Item-level Loevinger-tradition scores (multiple laboratories)	Feature/prompt discovery (fitness evaluation)	Yes (only dataset)
2 Cook-Greuter corpus	851 protocols	Protocol-level TPRs, single expert (Cook-Greuter)	Classifier calibration and accuracy evaluation (participant-level 10-fold CV)	No
3 Fournier et al. (2022)	446 participants	WUSCT scored by Fournier-laboratory raters	External construct and incremental validity	No
4 Pennebaker essays	2,468 participants	None (Big Five self-reports)	Theoretical pattern test	No
5 UBC/Suedfeld exemplars	155 texts	Expert Integrative Complexity scores	Language Complexity calibration/validation (10-fold CV)	No
6 Naturalistic IC corpus	2,344 texts	Trained-coder Integrative Complexity scores	Language Complexity calibration/validation (10-fold CV)	No
7 Lazar/Singleton cohort	47 protocols	Independent Cook-Greuter-trained scorer	Blinded replication	No

The evolutionary discovery phase had access only to Dataset 1; all indicator prompts and code-derived features were frozen before contact with any other dataset.

#### 2.1.1 Dataset for feature discovery (Loevinger corpus)

Dataset 1 served as the basis for the evolutionary algorithm's discovery of feature dimensions potentially underlying ego development stages. This dataset comprises the composite corpus described by Lanning et al. (2018), totaling 43,933 individual SCT item completions scored using Loevinger's framework and manual (Hy and Loevinger, 1996). It aggregates materials originally collected by Loevinger (n = 7,593 exemplar sentences), McAdams (*n* = 5,418), King (*n* = 4,364), and Lanning (*n* = 26,558). Item-level ratings span the full Loevinger range but concentrate in the conventional stages (E2: 418; E3: 1,751; E4: 10,171; E5: 15,667; E6: 12,139; E7: 3,194; E8: 579; E9: 14); ratings E4–E6 together comprise 86.4% of items, a point we return to in the Limitations. Given the aggregated nature of this corpus, consistent participant-level demographics and Total Protocol Ratings (TPRs) were not uniformly available. Its primary role was to provide broad text data against which the evolutionary algorithm could evaluate candidate scoring prompts, selecting those whose outputs best predicted the item-level stage labels (see Section 2.2 for details).

#### 2.1.2 Dataset for model evaluation (Cook-Greuter corpus)

Dataset 2 provided the criterion dataset for training the final classifier stage of the model (using features discovered from Dataset 1) and evaluating its stage scoring accuracy against expert judgment. This dataset comprises 30,636 SCT completions from 851 unique individuals, made available by Cook-Greuter. All responses were hand-scored at both the item level and for the overall protocol by Cook-Greuter, applying her refined scoring manual (Cook-Greuter, 1999; unpublished revisions). Dataset 2 labels were produced by a single expert scorer (Cook-Greuter), who serves as the criterion standard in EDT practice (current scorer certification programs typically target approximately 85% agreement with this standard; we therefore treat model-expert agreement as the primary validity metric here). The dataset includes these expert-assigned Total Protocol Ratings (TPRs), serving as the target variable for AI accuracy evaluation.

Participants were aged 25–65 years (*M* = 40.5, *SD* = 10.2), balanced in gender (52% female), and generally held at least a bachelor's degree. This dataset was the primary basis for assessing the model's core predictive performance.

#### 2.1.3 Dataset for external construct validation (Fournier corpus)

Dataset 3, the publicly available data from Fournier et al. (2022), was used to independently assess the construct validity of the AI-derived maturity indicators. This dataset includes *N* = 446 participants (53% female; age *M* = 48.39, *SD* = 7.30) who completed the Washington University Sentence Completion Test (WUSCT); two participants with null (missing) sentence-completion responses in the source data were excluded from indicator-based analyses, yielding an analytic sample of *N* = 444. It also contains measures of personality and personal coherence relevant to ego development. For our analyses, we focused on Big Five Agreeableness, Neuroticism, and Openness, the IPIP Unassuming-Ingenuous scale (Markey and Markey, 2009), the Author Composite score reflecting narrative coherence, the Index of Autonomous Functioning (IAF; Weinstein et al., 2012), and measures of Value-Behavior and Actor Coherence (Fournier et al., 2022). These coherence indicators span McAdams' (McAdams, 1995) three-layer personality framework: actor coherence (e.g., congruence among interpersonal traits, values, and behaviors), agent coherence (e.g., horizontal and vertical goal alignment, autonomous motivation), and author coherence (e.g., contextual, chronological, and thematic coherence in self-defining memories). We applied our AI model to the WUSCT responses to generate predicted EDT stages and maturity indicator scores for correlation with these external variables, and also utilized the expert-rated WUSCT scores available in this dataset for incremental validity analyses.

#### 2.1.4 Dataset for theoretical pattern validation (Pennebaker Essays Corpus)

Dataset 4, the Essays dataset collected by Pennebaker and King (1999), was used to test a specific theoretical prediction of EDT regarding alternating developmental phases. This dataset consists of 2,468 stream-of-consciousness texts from university students (60.3% female) who had also completed a Big Five personality inventory. Following an adaptation of the sentence-extraction approach of Lanning et al. (2018) we extracted SCT-like sentences (sentences longer than median length containing stems like “I am,” “My father/mother,” etc.) from the essays. Our AI model was applied to these extracted sentences to predict an EDT stage for each participant. Big Five scores (specifically Agreeableness and Extraversion) were binarized based on a median split of *z*-scores for analysis across predicted stages [only the binarized data was made available by Pennebaker and King (1999); the raw data is not available to the public].

#### 2.1.5 Datasets for integrative complexity validation

To calibrate and validate the *Language Complexity* Indicator, we utilized two independent, publicly available corpora scored for Integrative Complexity (IC). Using both an expert-anchored reference set and a large-scale set of naturalistic text allows for a robust test of the indicator's validity and its ability to generalize across different writing contexts.

Dataset 5: UBC/Suedfeld Expert Exemplars. The first corpus consists of 155 expert-scored paragraphs from the official University of British Columbia training materials curated by Suedfeld, as described by Conway et al. (2014). These texts serve as the reference standard for the 1–7 IC scale and were used to test the LC's performance against expert-defined ground truth.

Dataset 6: Naturalistic Online Discourse Corpus. The second corpus, from Jakob et al. (2023), provides a large-scale, real-world test set. We utilized the English-language subset of 2,344 online user comments (from news sites comments, Facebook, and Twitter) from their comparative study of public deliberation. Comments were scored for IC on the standard 1–7 scale by trained coding teams with high inter-rater reliability (Krippendorff's α = 0.85 − 0.88). Unscorable comments (IC = 0) were excluded from our analyses.

#### 2.1.6 Dataset for independent blinded replication (Lazar/Singleton Corpus)

Dataset 7 comprises *N* = 47 de-identified SCT protocols (15% post-conventional) drawn from a meditation-research cohort (Singleton et al., 2021). Human reference scores (TPR and TWS) were produced by a scorer trained by Cook-Greuter & Associates (Wayland, MA, United States). The automated scoring system and the human scorer were fully blinded to each other's outputs. This dataset also includes MSCEIT scores (Mayer–Salovey–Caruso Emotional Intelligence Test), enabling validation of the Emotional Integration indicator against a performance-based measure of emotional reasoning ability. Study procedures and numerical outcomes were independently verified and signed by Assoc. Prof. Sara W. Lazar (Harvard Medical School/Massachusetts General Hospital).

#### 2.1.7 Ethical considerations

All data analyzed in this manuscript were archival, sourced from datasets collected under protocols approved by the institutional review boards at the respective original data collection institutions. Informed consent was obtained from all participants during the original data collection phases. The current study involved secondary analysis of anonymized data and adhered to all relevant ethical guidelines for research. All processing of participant data was performed using a self-hosted language model deployed on infrastructure certified as SOC 2 Type II, GDPR, and HIPAA compliant. No participant data was transmitted to any public or third-party LLM service (e.g., ChatGPT, Claude, or similar platforms); the frontier-model baseline evaluations (Section 2.4.2) used only de-identified exemplar material derived from the scoring manual, submitted via the OpenAI API under its standard no-training, limited-retention data policy.

### 2.2 The evolutionary AI model for feature discovery

To overcome the limitations of direct manual encoding or simplistic LLM prompting, and inspired by the premise that EDT reflects underlying structures in meaning-making (Kegan, 1982; Cook-Greuter, 1999), we developed an approach that discovers candidate dimensions predictive of EDT stage directly from data. This involved three main steps: establishing a capable foundation language model, and evolving prompts and language complexity metrics defining potential “maturity indicators” using a genetic algorithm, and calibrating the language complexity metrics on a labeled dataset.

We selected an evolutionary approach (a genetic algorithm variant; Vikhar, 2016; Lambora et al., 2019) over conventional supervised learning for two reasons. First, the relationship between linguistic features and EDT stage is complex and non-linear; for example, deliberate simplicity—“simplicity on the other side of complexity” (attributed to Oliver Wendell Holmes Sr.)—can be a hallmark of the latest-stage responses. Second, we required interpretable features that align with psychological theory rather than arbitrary statistical patterns.

#### 2.2.1 Foundation language model

We utilized a Large Language Model (LLM) with 72B parameters based on the Qwen2.5 architecture (Yang et al., 2024), selected as the most capable open-weights model available at the time of development that could be deployed on self-hosted, compliance-certified infrastructure (a data-protection requirement; see Section 2.1.7). The base model was adapted in two phases using low-rank adaptation (LoRA, rank 16; supervised phase: 3 epochs, learning rate 2 × 10^−4^; preference phase: 3 epochs, learning rate 5 × 10^−5^, β = 0.1, effective batch size 4, linear schedule, 8-bit AdamW). First, supervised fine-tuning used exclusively general-purpose, publicly available instruction-tuning collections containing no ego-development-related content. Second, preference tuning (Odds Ratio Preference Optimization; Hong et al., 2024) used 14,264 preference pairs constructed from the exemplar pool of Cook-Greuter's scoring manual and from publicly available research papers authored by, or citing, Cook-Greuter: for each manual exemplar, the preferred response reflected the manual-consistent stage interpretation, while the rejected response was sampled from the most semantically similar exemplar (by cosine similarity) belonging to an adjacent stage. Interim evaluation showed that domain adaptation alone, like manual-based prompting of frontier models, does not yield expert-level stage agreement (59.2% direct-prediction exact agreement; reported as an ablation in Section 3.1)—converging evidence that knowledge of the scoring manual is not sufficient for this task, and the design rationale for the indicator-discovery approach described next.

#### 2.2.2 Evolutionary prompt optimization

We employed a Genetic Algorithm (GA; Lambora et al., 2019) framework for LLM-driven prompt evolution over a frozen scorer model, following the EvoPrompt paradigm (Guo et al., 2024) (cf. EvoPrompting, which evolves architectures with per-generation retraining; Chen et al., 2023). The adapted LLM remained frozen throughout evolution; only the prompt population evolved. The evolved prompts are anchored scoring rubrics: each instructs the frozen LLM to analyze an SCT response and output a score reflecting a specific potential facet of ego development. We refer to these individual rubrics as sub-indicators; each of the named “maturity indicators” described below comprises several conceptually related sub-indicators.

- *Population and evolution:* The GA operated on a population of candidate prompts. Initial prompts were seeded by the four conceptually distinct but developmentally coherent domains identified by Loevinger—character (impulse control and self-regulation), interpersonal style, conscious preoccupations, and cognitive style (Loevinger, 1976; Binder, 2023)—and subsequently diversified through the evolutionary process. The LLM itself executed mutation and crossover operations via specifically designed meta-prompts, iteratively refining the prompt population over generations. Evolution continued until fitness improved less than 1% over 10 consecutive generations.
- *Fitness function:* The fitness of a prompt set (representing a potential feature space) was evaluated based on its ability to predict expert-assigned, item-level EDT scores within the large discovery dataset (Dataset 1 - *Loevinger Corpus*). Specifically, fitness was defined by the classification accuracy of a multinomial logistic regression model trained to predict item-level stage scores using the LLM-generated scores from all currently evolved rubrics as input features.
- *Adaptive population sizing:* the population was regularly pruned, excluding features not significantly associated with stage of ego development, as well as features whose removal did not reduce fitness. This enforces conditional non-redundancy—each retained feature contributes incremental predictive value—rather than pairwise independence; the retained indicators remain substantially intercorrelated (Supplementary Appendix B).

See Supplementary Appendix C for more details about the genetic algorithm framework employed to discover effective prompts.

A single set of maturity indicator prompts was applied uniformly across all 36 sentence stems. We opted for stem-agnostic scoring because the indicators are designed to capture structural features of meaning-making (e.g., integrative complexity, perspective-taking capacity) rather than content-specific themes, and because stem-specific models would have fragmented the training data and complicated generalization. Importantly, the entire evolutionary discovery process used only Dataset 1 (Loevinger Corpus); no design choices—including the number of indicators, stopping criteria, or fitness function—were informed by performance on Dataset 2. The resulting prompts were frozen before any evaluation on Cook-Greuter-scored data.

The final set of “maturity indicators” discovered—each comprising several conceptually related sub-indicator rubrics—included (see Supplementary Appendix A for descriptions): **Relational Maturity, Emotional Integration, Value-Driven Action, Self-Directed Leadership, Ethical Alignment, Holistic Thinking, Societal and Cultural Awareness, and Self-Transcendent Awareness**. An additional indicator, **Language Complexity**, was included because the structural complexity of language—how ideas are differentiated and integrated syntactically—is theoretically central to EDT (Loevinger, 1976; Lanning et al., 2018) and empirically associated with stage across scoring traditions, yet is not directly captured by LLM-rated semantic rubrics.

#### 2.2.3 Addition of the language complexity indicator

The Language Complexity (LC) indicator was developed using a two-stage process distinct from the other eight indicators. Instead of evolving scoring rubric prompts, we used an evolutionary algorithm to generate novel, executable Python (Python Software Foundation, Wilmington, DE, United States) functions that could capture the nuances of linguistic complexity predictive of ego development.

**Stage 1. Evolutionary discovery of code-based complexity features**. The process began by seeding the evolutionary algorithm with a population of prompts. These prompts contained natural language descriptions of established linguistic complexity metrics drawn from computational linguistics literature. The concepts included:

- Morpho-syntactic signals (e.g., part-of-speech distributions, type-token ratio).
- Syntactic features (e.g., dependency relations, parse-tree depth).
- Markers of differentiation and integration (e.g., syntactic subordination, concessive/causal connectives, lexical diversity).

Within the evolutionary loop, the fine-tuned LLM's task was to act as a code generator, translating these evolving natural language descriptions into executable Python functions. The fitness of each generated function was determined by how well its numerical output correlated with the human-rated ego development stages in Dataset 1. This approach allowed the algorithm to not only select but also to modify and synthesize entirely new programmatic features optimized for predicting psychological maturity from text.

**Stage 2. Calibration against integrative complexity**.

The set of high-performing, evolutionarily-derived Python features from Stage 1 was then used to create a single, robust LC score. To do this, we trained a final linear regression model using these features as predictors. The model was trained to predict human-rated Integrative Complexity (IC) scores, using the expert-scored corpora from Datasets 5 and 6 as the ground truth.

This final calibration step ensures that our discovered linguistic features are anchored to the well-established construct of Integrative Complexity, resulting in a single, validated Language Complexity indicator.

#### 2.2.4 Inference pipeline

At inference time, each of the 36 stem completions in a protocol is scored independently by the frozen LLM. Each of the eight LLM-derived maturity indicators comprises several evolved sub-indicator rubrics; each rubric instructs the model to rate a completion on a 1–10 scale with anchored band descriptions. For example, one evolved sub-indicator rubric reads:

**Intrinsic motivation:** The degree to which the sentence completion reflects motivation driven by internal factors such as personal growth, curiosity, or a sense of purpose, rather than external rewards or incentives.

1–3: Primarily driven by external rewards, status, or material gain.

4–6: Balances intrinsic fulfillment with practical needs and societal expectations.

7–10: Transcends reward systems, acts from pure intrinsic motivation, compassion, or universal principles.

A second example, illustrating a reverse-scored rubric, appears in Supplementary Appendix C. Sub-indicator ratings are averaged across the 36 completions, yielding protocol-level sub-indicator scores; this vector—comprising all 75 sub-indicator scores—serves as input to the multinomial logistic classifier, which outputs the predicted stage. The Language Complexity features are computed programmatically as described above and aggregated across completions in the same way. For interpretation and validation, sub-indicator scores are additionally averaged within each indicator, yielding the nine maturity indicator scores used throughout the Results.

### 2.3 Safeguards against overfitting and information leakage

Evolutionary prompt optimization is a highly flexible procedure, and flexible procedures can capitalize on corpus-specific regularities. We therefore implemented, and document here, explicit safeguards against overfitting and information leakage; Table 1 summarizes which dataset was exposed to which phase of the pipeline.

1. *Single-exposure discovery*. The entire evolutionary loop—population initialization, fitness evaluation, pruning, and stopping—interacted exclusively with Dataset 1. The number of indicators, the stopping criterion, and the fitness function were fixed before any contact with Datasets 2–7, and the resulting indicator prompts and code-derived features were frozen thereafter.
2. *Adaptation-corpus containment*. The two adaptation phases deliberately separated general capability from domain specialization. Supervised fine-tuning used exclusively publicly available, general-purpose corpora—approximately 2.6 GB across 14 components spanning instruction-following and conversational data (the majority), programming and mathematical-reasoning data (a standard element of instruction mixtures that improves general reasoning performance; Ma et al., 2024), and tool-use data, together with a small synthetic conversational corpus including psychology- and therapy-style dialogue (≈0.2% by size); no component contains ego-development, sentence-completion, or stage-scoring content. Domain-specific material entered only through the preference-optimization phase, which used Cook-Greuter's scoring manual—including its scored exemplar completions—and publicly available EDT literature. Scored manual exemplars were therefore part of the adaptation corpus; however, no responses from any evaluation dataset were included: Datasets 2 and 7 consist of private, never-published protocols that appear in no training source, and none of the public evaluation corpora (Datasets 3–6) were part of the adaptation data. The model's relationship to the scoring manual thus parallels that of a certified human scorer, who likewise trains on manual exemplars before scoring novel protocols; notably, frontier models given the identical manual in-context reached only 40.1%–44.9% accuracy, and the manual-adapted model itself, scoring directly without the evolved indicators, reached only 59.2% (Section 3.1)—indicating that access to manual content, whether in-context or through adaptation, does not by itself produce the reported performance. To verify the absence of item-level overlap directly, we performed a string-level check between the exemplar pool underlying the 14,264 preference pairs (17,369 exemplar sentences) and the 30,636 Dataset 2 completions (29,934 non-empty). After normalization (case and whitespace), 354 completions (1.18% of non-empty completions; 238 of them verbatim matches) share their text with an exemplar, of which 248 (0.83%; 208 unique strings) also carry the same assigned stage. Matched strings are characteristically short, generic completions (median 8 words including the stem, vs. a corpus median of 15) of the kind that independent respondents produce coincidentally, and identical wording receiving identical stages is expected under any consistent application of the scoring manual; string identity therefore cannot establish shared provenance. As an upper bound, even treating all 248 stage-concordant matches as shared items, their influence is limited by a unit mismatch: the matches are completion-level, whereas the accuracy criterion is protocol-level, with each prediction aggregating 36 completions (no protocol contains more than five matched completions). Testing directly for any residual advantage, protocols containing at least one matched completion were scored no more accurately than protocols containing none (85.8% vs. 86.2%; *n* = 148 vs. 703; Fisher exact *p* = 0.90)—not the elevated accuracy that contamination would produce. The direct-scoring ablation (Section 3.1) provides a further independent bound on what adaptation-phase exposure can explain.
3. *Frozen, verifiable artifact*. All inference was deterministic (temperature = 0, fixed seeds), and the frozen inference artifact is identified by a published SHA-256 digest (see Section Data availability statement), providing a tamper-evident reference for verification.
4. *Participant-level cross-validation*. On Dataset 2, only the final multinomial logistic classifier was refit within each training fold; the indicator definitions remained fixed. Folds were stratified by expert TPR, and the same person never appeared in both training and test folds.
5. *No tuning on evaluation data*. No hyperparameter of the discovery process was revisited after observing performance on Dataset 2.
6. *Held-out external datasets*. Datasets 3, 4, and 7 were scored once with the frozen model, without any refitting. Datasets 5 and 6 were used solely to calibrate the Language Complexity regression, under their own cross-validation.
7. *Independent blinded replication*. Dataset 7 was scored by an independently trained human rater and by the model under mutual blinding, with procedures and numerical outcomes verified by an independent third party.

These safeguards rule out direct leakage, but they cannot rule out subtler forms of capitalization: Datasets 1 and 2 share a genre (SCT completions) and partially similar populations, so features exploiting genre- or population-specific regularities could in principle survive the dataset separation. For this reason, we place particular weight on validation against criteria that share neither genre nor scorer with the training pipeline—the external construct validity, behavioral, and non-SCT analyses reported below.

### 2.4 Benchmarking and validation procedures

The following procedures were employed to evaluate the AI model's performance against human benchmarks and validate its outputs against theoretical expectations and external data.

#### 2.4.1 Human performance benchmarking

To establish a comparative baseline for the AI model's accuracy, we referenced established benchmarks for inter-rater reliability (overall stage agreement as percentage exact match) among expert human scorers. For Cook-Greuter's system, we primarily drew upon rates reported around 85% by Cook-Greuter (1999) and compared with rates from other contexts (e.g., Torbert and Livne-Tarandach, 2009). Comparisons were also made to typical reliability reported for Loevinger's system, including recent findings (e.g., Fournier et al., 2022).

#### 2.4.2 AI model performance evaluation

The model's core accuracy in predicting expert-assigned TPRs was evaluated on Dataset 2 (Cook-Greuter Corpus) using a 10-fold stratified cross-validation procedure, ensuring the same person never appears in both train and test folds. Stratification by expert TPR ensured that each fold reflected the original distribution of EDT stages. Performance was assessed using:

- Overall classification accuracy (percentage exact match with expert TPR), and quadratic-weighted Cohen's κ to quantify chance-corrected agreement on an ordinal scale. We report 95% confidence intervals (CIs) computed on participant-level, out-of-fold predictions using a bootstrap procedure (5,000 resamples).
- Per-stage Precision, Recall, F1-Score
- A detailed confusion matrix visualizing classification patterns between stages.

To evaluate the AI model against contemporary alternatives, we also assessed the zero-shot performance of two frontier LLMs (OpenAI's GPT-4o and o3, specific versions “gpt-4o-2024-08-06” and “o3-2025-04-16”) on the same scoring task, providing the models with SCT responses and the scoring manuals via direct prompting (using the verbatim prompt “Score this SCT—determine stage of ego development—according to Cook-Greuter's system. Only output stage and nothing else. Guide:\n,” followed by the scoring manual).

Furthermore, to examine the structure emerging within the discovered feature space (composed of the evolved sub-indicator scores), we applied two dimensionality-reduction techniques to visualize participant responses from Dataset 2: Linear Discriminant Analysis (LDA), a supervised projection that maximizes separation between expert-scored stages, and Uniform Manifold Approximation and Projection (UMAP), a nonlinear technique used here purely for visualization. The quality of stage-based clustering within this space was quantitatively assessed using the Calinski-Harabasz (CH) Index and the Rand Index, comparing cluster assignments derived from *k*-Means clustering against expert-assigned stages. We treat these low-dimensional projections as descriptive visualizations of the learned feature space rather than as tests of developmental topology; supervised projections in particular can induce apparent trajectory-like geometry (see Section 3).

#### 2.4.3 External validation analyses

We conducted validation analyses using two independent datasets (Datasets 3 and 4) to assess the psychological meaningfulness and theoretical alignment of the AI model's outputs.

##### 2.4.3.1 Alternating pattern validation (Dataset 4)

To test EDT's theoretical pattern of alternating differentiation/integration stages (Cook-Greuter and Soulen, 2007), we first applied the finalized AI model to predict EDT stages from SCT-like sentences extracted from the Pennebaker Essays Corpus (Dataset 4). We then compared the means of binarized Big Five Agreeableness and Extraversion scores across adjacent AI-predicted stages using non-parametric Mann–Whitney *U* tests to detect the expected alternating pattern.

##### 2.4.3.2 Construct and incremental validity (Dataset 3)

Using the Fournier et al. (2022) dataset (Dataset 3), we applied the finalized AI model to the participants' WUSCT responses to generate predicted EDT stages and scores on the nine maturity indicators

- *Correlational analysis:* We calculated Spearman's Rho correlations between the AI-derived maturity indicator scores and theoretically relevant external variables available in Dataset 3 (Big Five Agreeableness, Neuroticism, and Openness; IPIP Unassuming-Ingenuous; Author Composite; IAF Index; and Coherence indices) to assess convergent and discriminant validity patterns.
- *Incremental validity analysis:* We performed Hierarchical Multiple Regression (HMR) analyses predicting key external variables (Agreeableness, Neuroticism, Coherence indices, and IAF) and the expert-rated WUSCT stage scores also present in Dataset 3. Model 1 included the five Big Five traits as predictors; Model 2 added the nine AI maturity indicators. The significance of the change in *R*-squared (Δ*R*^2^) was examined to determine the incremental variance explained by the AI indicators beyond broad personality traits.

#### 2.4.4 Linguistic complexity comparison

Language Complexity consisted of complexity metrics suggested by the evolutionary algorithm, combined using a regression model.

The validation strictly separated the two stages of the indicator's development to prevent data contamination: Stage 1 involved discovering potential linguistic features using only the EDT-scored Loevinger corpus (Dataset 1), with no exposure to the Integrative Complexity (IC) datasets. In Stage 2, the best features from the first stage were used as fixed inputs to a final regression model calibrated exclusively on the human-rated IC scores from Datasets 5 and 6.

Performance was assessed using a 10-fold cross-validation procedure on the pooled IC corpora (Datasets 5 and 6). The reported Krippendorff's α reflects the internal consistency between the vector of our model's scores and the vector of human expert scores across all held-out texts. The strength of the association between Language Complexity (LC) and (i) overall EDT stage and (ii) external psychometric variables (e.g., personality scales) was quantified with Spearman's rank correlation (ρ). Spearman's ρ was chosen because EDT stage is ordinal with many ties, several variables exhibited non-normal and heteroscedastic distributions, and relationships were expected to be monotonic rather than strictly linear. Ties were handled via average ranks. Where *p*-values are reported, they are two-sided and adjusted for multiple comparisons within each family of tests using Benjamini–Hochberg FDR.

Controlling for verbosity/readability: To test whether the LC indicator predicts EDT stage beyond verbosity, we ran two complementary analyses on the full sample (*n* = 851). First, we computed a partial Spearman correlation between LC and stage while controlling for log(words + 1) (and, in a separate analysis, Flesch–Kincaid grade, calculated by the Python package textstat), implemented via rank-residualization with 5,000 bootstrap resamples to form 95% CIs. Second, we fit proportional-odds ordinal logistic models with stage as the outcome and standardized LC plus one control covariate (either log(words + 1) or Flesch–Kincaid grade) as predictors. We report odds ratios (OR) per 1 SD increase in LC with Wald 95% CIs and a likelihood-ratio (LR) test against a control-only model.

## 3 Results

This section presents the empirical findings in the order of the three claims distinguished in the Introduction: scoring performance against human benchmarks and baselines (measurement automation), external validation of the indicators (construct validity), and analyses bearing on linguistic complexity and the organization of the feature space (structural interpretation).

### 3.1 AI model performance on EDT scoring

The model's capacity to predict expert-assigned Cook-Greuter EDT stages (TPRs) was evaluated using 10-fold stratified cross-validation on the expert-rated Dataset 2 (Cook-Greuter Corpus).

#### 3.1.1 Overall accuracy and benchmark comparisons

The model achieved an overall accuracy of **86.1%** (95% CI [83.8, 88.4]) in predicting the exact expert-assigned TPR (Spearman's ρ = 0.937; quadratic-weighted Cohen's κ = 0.927, 95% CI [0.912, 0.941]). This performance is comparable to established benchmarks for inter-rater reliability among trained expert scorers using Cook-Greuter's system, typically reported around 85% (Cook-Greuter, 1999). Agreement levels vary with context [e.g., 72% reported by Torbert and Livne-Tarandach (2009) for scorers not trained by Cook-Greuter]; our model reaches the standard cited for scorers trained directly by the system's developer, and is within the range reported for Loevinger's earlier system (60%–86%; Fournier et al., 2022 report 79%). One asymmetry of this comparison should be noted: our classifier was calibrated to the criterion scorer's labels (within cross-validation), whereas published human–human agreement rates reflect two independently trained raters. The blinded replication reported below, in which the model's predictions were compared with those of an independently trained scorer on a new sample, provides the more direct analog of the human certification scenario and yielded a comparable figure (85.1%). Given the concentration of observations at Stages 4 and 4/5, imbalance-appropriate chance references further contextualize these figures: a majority-class predictor attains 36.3% exact agreement and a distribution-matched random predictor 27.2%, compared with the model's 86.1% (the quadratic-weighted κ reported above is chance-corrected).

We also assessed the zero-shot performance of two frontier language models on the same task: OpenAI's gpt-4o-2024-08-06 and the reasoning-optimized o3-2025-04-16. Provided with the raw SCT responses and the scoring manual via direct prompting, these models reached overall accuracies of 40.1% and 44.9%, respectively, with maximum stage errors of 4 and 3 full stages and standard deviations of differences of 0.817 and 0.651. These figures align with the initial prompting experiments of Bronlet (2025), who observed differences of up to 3 full stages and standard deviations between 0.63 and 0.98. Their subsequently refined protocol (prompt refinement with median aggregation over repeated runs) reports a weighted κ of 0.78 for single sentences on a 58-sentence test set, and of 0.71 for scores aggregated over 10-sentence combinations, using scoring dimensions derived from a different developmental model (O'Fallon's STAGES). These results are not directly comparable to the protocol-level, full-instrument agreement examined here.

For completeness, we also evaluated two simple baselines: a bag-of-words model (35.8% accuracy) and a classifier over state-of-the-art sentence embeddings (voyage-3-large, which led the Massive Text Embedding Benchmark at the time of writing; 36.7% accuracy).

As a further ablation, we evaluated the domain-adapted LLM itself as a direct stage scorer, without the evolved indicators. Prompted to assign stages directly, it reached 59.2% exact agreement with expert TPRs across all 851 protocols (95% CI [55.9, 62.5]; the adapted model had no contact with Dataset 2 during adaptation, Section 2.3)—above frontier models prompted with the full manual (40.1–44.9%), but well below both expert inter-rater levels (≈85%) and the full indicator-based system (86.1%). Domain adaptation alone therefore accounts for only a modest share of the reported performance; the decisive contribution comes from the evolved indicators.

**Figure 1:**
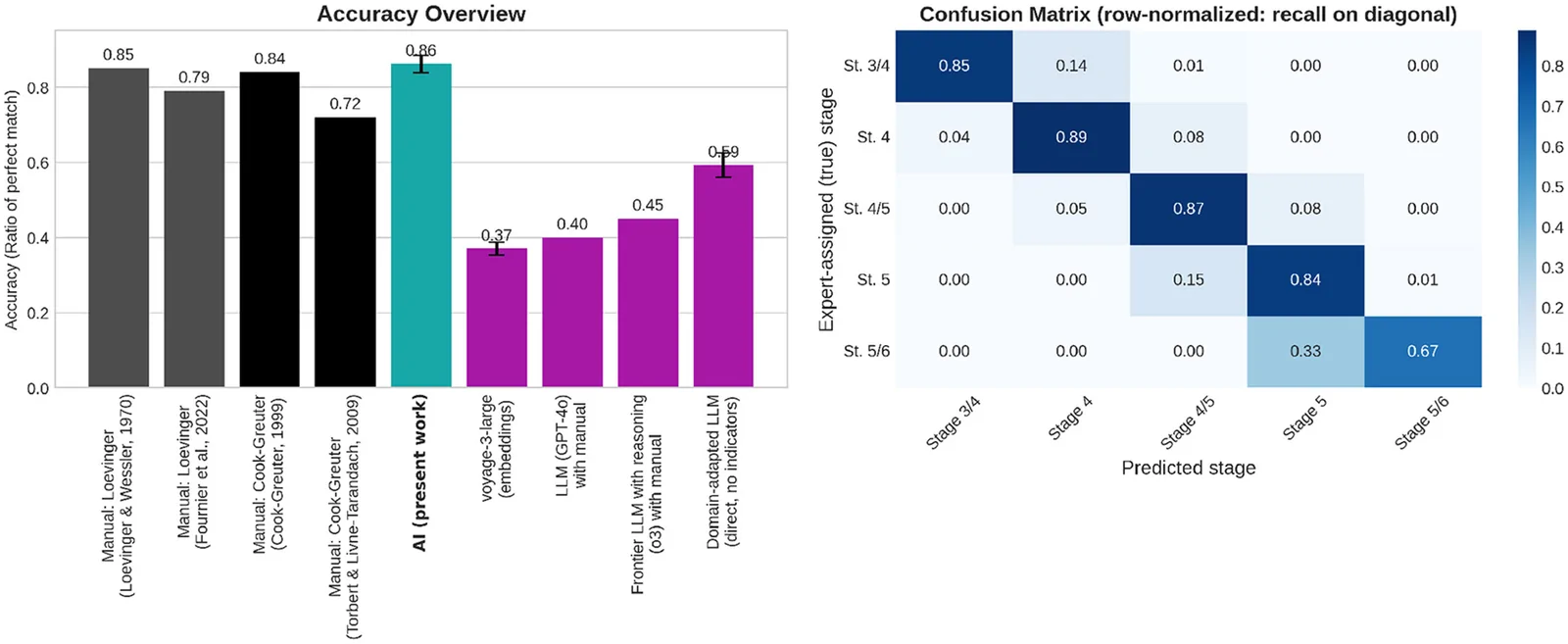
Predictive accuracy of the AI model compared with human inter-rater benchmarks and baselines. **(Left)** percentage exact-match agreement for human scorers using Loevinger's older scoring system (bars 1 and 2, gray) and Cook-Greuter's newer system (bars 3 and 4, black), the AI model (teal bar), and baseline methods (magenta bars), including the domain-adapted LLM scoring directly without the evolved indicators (rightmost bar; ablation, Section 3.1). Error bars are bootstrap 95% CIs where available. **(Right)** confusion matrix of expert-assigned (rows) vs. AI-predicted (columns) stages, row-normalized so that the diagonal shows per-stage recall (cf. Table 2).

Figure 1 (left panel) contrasts the performance of our model with human benchmarks and these baselines. The pattern indicates that expert-comparable stage scoring is not achieved by prompting general-purpose models with scoring manuals, and that the evolutionary feature discovery contributes materially to performance. Methods that reflect only content and word choice (bag-of-words, embeddings) similarly failed to capture the structure of meaning-making.

#### 3.1.2 Per-stage performance and confusion patterns

Table 2 details the model's performance for each EDT stage present in Dataset 2. Precision and recall were generally high (with balanced accuracies mostly around 0.9), particularly for the more populated conventional stages (Stage 4 and 4/5). Performance was slightly lower for the later, less frequent stages (Stage 5 and 5/6), reflecting the inherent difficulty in distinguishing these nuanced levels of development.

**Table 2:** Per-stage performance metrics from 10-fold cross-validation.

Stage	Precision	Recall	F1-score	Balanced accuracy	*N*
3/4	0.879	0.853	0.866	0.918	102
4	0.910	0.887	0.898	0.918	309
4/5	0.827	0.869	0.847	0.895	259
5	0.809	0.841	0.825	0.899	151
5/6	0.909	0.667	0.769	0.832	30
Overall (accuracy)	**86.1%** (95% CI [83.8, 88.4])				851
Overall quadratic-weighted Cohen's κ	**0.927** (95% CI [0.912, 0.941])				851

Bold values denote overall performance aggregated across all stages.

The confusion matrix, presented in Figure 1 (right panel), confirms this pattern. As expected, the vast majority of misclassifications occurred between immediately adjacent stages (e.g., predicting Stage 4/5 when the true stage was 4, or vice versa). Significant confusion across non-adjacent stages was rare. This pattern aligns with the continuous nature of development and the challenges human scorers also face distinguishing adjacent levels. The slight drop in F1 for Stage 5/6 is also influenced by its lower base rate (*N* = 30) in this dataset.

### 3.2 Independent blinded replication

To assess generalization beyond the primary training corpus and criterion scorer, we conducted a blinded comparison on an independent sample of 47 SCT protocols collected as part of a meditation-research program (Singleton et al., 2021). Human reference scores were produced by a scorer trained by Cook-Greuter & Associates—distinct from the single expert whose ratings comprise Dataset 2. Neither the human scorer nor the automated system had access to each other's outputs. Results closely replicated the primary evaluation: exact-match stage accuracy was 85.1% (40/47 protocols correct) and the correlation between model-predicted and human-assigned Total Weighted Scores was *r* = 0.963. These figures are consistent with the cross-validated estimates from Dataset 2 (86.1% accuracy, κ_*w*_ = 0.927), suggesting that model performance generalizes across an independent rater and sample within the Cook-Greuter scoring tradition, including protocols at post-conventional stages. Because scorer and model were trained independently and blinded to each other, this comparison is structurally equivalent to the inter-rater agreement assessed in human scorer certification, and therefore constitutes our most direct evidence of human-comparable scoring.

### 3.3 Structure within the discovered feature space

**Figure 2:**
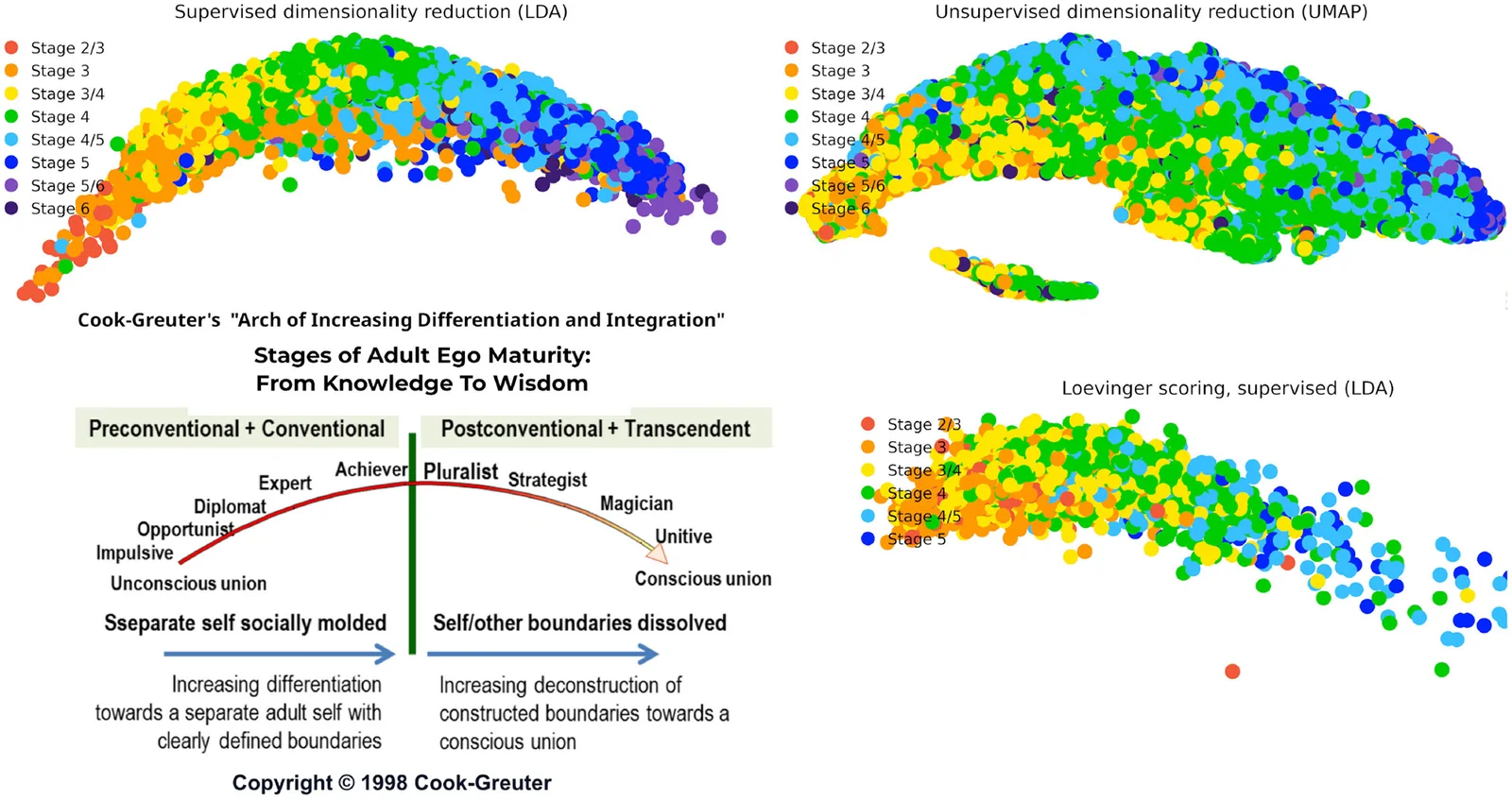
Dimensionality-reduced visualization of SCT completions in the AI-derived feature space, shown for descriptive purposes. **(Top left)** Dataset 2 (Cook-Greuter scored) with supervised dimensionality reduction (LDA); colors correspond to EDT stages. **(Bottom left)** theoretical model of the developmental trajectory (© S. Cook-Greuter, reproduced with permission; cf.Cook-Greuter, 1999), shown for qualitative comparison. **(Top right)** Dataset 2 with unsupervised dimensionality reduction (UMAP). **(Bottom right)** Dataset 1 (Loevinger scored). Note that LDA is supervised by stage labels, and that curvilinear (arch-shaped) geometries can arise artifactually in low-dimensional embeddings of ordinal data; these projections illustrate the feature space and are not evidence of developmental topology (see text).

The evolutionary algorithm was designed not just to predict stage but to discover a feature space reflecting developmental structure. Figure 2 visualizes participant SCT responses embedded within this space, reduced using Linear Discriminant Analysis (LDA) supervised by expert-assigned item scores for each stem completion. Clustering is evident in both the Cook-Greuter scored data (Dataset 2, top) and the Loevinger scored data (Dataset 1, bottom right), suggesting the discovered features capture developmental distinctions common to both scoring traditions. The arrangement of stages forms a curvilinear trajectory (top panels) that is visually reminiscent of the theoretical path of development proposed by Cook-Greuter (1999), Cook-Greuter (2004) (bottom left); we interpret this correspondence cautiously below.

Quantitative indices support the visual clustering. The Calinski-Harabasz (CH) Index, measuring the ratio of between-cluster to within-cluster variance using expert-assigned stages in Dataset 2 as labels, was 121.8, indicating strong separation, and unsupervised *k*-Means clustering (with *k* set to the number of observed stages) yielded clusters substantially congruent with expert-assigned stages (Rand Index = 74.7%).

Two caveats constrain the interpretation of this geometry. First, the LDA projection is supervised by stage labels, so clear stage separation along the first discriminant is expected whenever the features carry stage-relevant information; it cannot serve as independent evidence of developmental ordering. Second, curvilinear “arch” or horseshoe shapes are a well-documented artifact of low-dimensional embeddings of data with an underlying ordinal gradient (Kendall, 1971; Diaconis et al., 2008), and UMAP geometries are additionally sensitive to hyperparameter choices. The inverted-U trajectory in Figure 2 is therefore *consistent with* Cook-Greuter's theorized developmental arc—an initial strengthening and differentiation of self-boundaries peaking around a stage of maximal self-definition, followed by a gradual loosening and re-integration of self-other boundaries (Cook-Greuter, 1999; Cook-Greuter and Soulen, 2007)—but cannot confirm it. What the projections do convey descriptively is twofold. First, stages occupy largely contiguous, ordered regions of the feature space, with adjacent stages neighboring one another. Second, the discriminant loadings are interpretable: the first discriminant reflects overall developmental maturity, with all maturity indicators loading in the same direction, while the second is dominated by Holistic Thinking (positive) and Societal & Cultural Awareness (negative), capturing the relative balance between these two forms of complexity across stages—a balance that peaks at the Self-Determining stage (Stage 4) and declines thereafter as sociocultural awareness accelerates. This descriptive pattern aligns with, but does not test, Cook-Greuter's claim that the conventional-to-post-conventional transition involves a reorientation from operating within a self-authored identity toward an increasing capacity for perspectival and contextual integration.

All eight LLM-derived indicators increase monotonically with expert-assigned item-level stage ratings (see Supplementary Appendix A), with the steepest differentiation occurring at different points along the developmental continuum, consistent with the multidimensional account developed above.

### 3.4 External validation

Beyond classification accuracy, we assessed whether the AI model's outputs capture psychologically meaningful variance related to established theoretical patterns and external constructs, using independent datasets.

#### 3.4.1 Evidence for alternating differentiation/integration pattern (Pennebaker Dataset)

**Figure 3:**
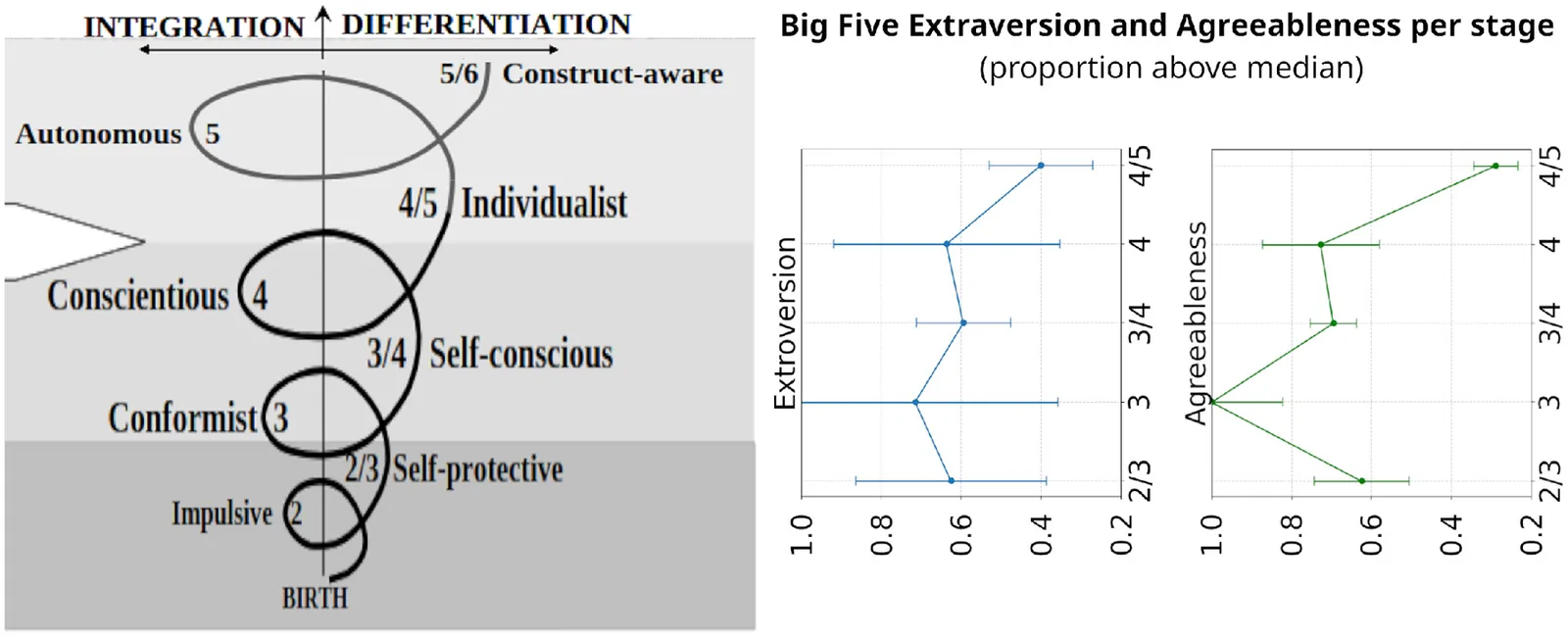
**(Left)** theoretical model of alternating stages of differentiation and integration in EDT (adapted from Cook-Greuter and Soulen, 2007). **(Right)** mean binarized Agreeableness and Extraversion scores (error bars are Wilson score intervals) across EDT stages in the Pennebaker essays data (Dataset 4, *N* = 2,468). Stages were predicted by the model from extracted SCT-like sentences and are not expert-rated. The alternating pattern across adjacent stages, clearest for Agreeableness, is consistent with the theoretical model shown at **(left)**.

EDT posits that development progresses through alternating phases emphasizing either differentiation (autonomy, separation) or integration (connection, relatedness) (Cook-Greuter and Soulen, 2007). We tested this by applying our AI model to SCT-like sentences extracted from the large Pennebaker stream-of-consciousness dataset (*N* = 2,468 students) and examining mean Big Five scores across AI-predicted EDT stages. Theoretically, differentiation phases might manifest as lower Agreeableness or higher Extraversion, while integration phases might show the opposite. As depicted in Figure 3, the analysis revealed a zig-zag pattern consistent with this theory, particularly for Agreeableness. Mann–Whitney *U* tests confirmed significant shifts between adjacent stages in the predicted directions: for example, lower Agreeableness from Stage 2/3 (integration) to Stage 3 (differentiation) (*U* = 182,103, *p* = 0.003), higher Agreeableness from Stage 3 to 3/4 (integration) (*U* = 293,641.5, *p* = 0.042), and lower Agreeableness again from Stage 4 (integration) to 4/5 (differentiation) (*U* = 6,036, *p* = 0.024). Similar, though less consistent, patterns were observed for Extraversion (e.g., a significant increase from Stage 2/3 to 3; *U* = 182,511, *p* = 0.003). These findings are consistent with the alternating-phase hypothesis, though the evidence should be interpreted cautiously: stages were AI-predicted rather than expert-rated, Agreeableness and Extraversion are indirect proxies for differentiation and integration, and the extracted sentences are not equivalent to standard SCT protocols. We accordingly regard this analysis as exploratory and hypothesis-generating.

#### 3.4.2 External construct and incremental validity (Fournier et al. Dataset)

To further evaluate the construct validity of the AI-derived maturity indicators, we leveraged the independent dataset published by Fournier et al. (2022) (*N* = 446). This dataset allowed examination of the relationships between the nine maturity indicators (derived as described in the Section 2) and theoretically relevant external variables assessed by Fournier and colleagues. We focused primarily on established personality traits capturing interpersonal style, adjustment, and intellectual engagement (Big Five Agreeableness, Neuroticism, Openness; IPIP Unassuming-Ingenuous), indices reflecting personality coherence and integration (Author Composite, Value-Behavior Coherence, and Actor Coherence), and autonomous functioning (IAF Index), all constructs conceptually linked to facets of ego development.

First, we assessed convergent and discriminant validity by examining zero-order correlations (Spearman's ρ) between the nine AI maturity indicators and selected external variables. As detailed in Table 3, the observed associations align with theoretical expectations for measures of psychological maturity. Overall, the indicators showed predominantly positive relations with adaptive traits (Big Five Agreeableness; prosocial IPIP Unassuming-Ingenuous), indices of personality coherence (value-behavior, actor), and Autonomous Functioning (IAF), with generally small or non-significant associations with Neuroticism—consistent with these indicators capturing developmental maturity rather than inverse emotional distress. Agreeableness effects ranged from small to moderate (ρ ≈ 0.14–0.41), coherence from small to moderate (ρ ≈ 0.09–0.40), and IAF from small to moderate (ρ ≈ 0.07–0.28).

**Table 3:** Correlations (Spearman's Rho) between AI maturity indicators and external variables from Fournier et al. (2022) Dataset (analytic *N* = 444).

Indicator	Big5	Big5	Big5	IPIP	Author	Autonomous	Value-	Actor	WU
	Agreeableness	Neuroticism	Openness	Unassuming-Ingenuous	Composite	Functioning Index (IAF)	Behavior Coherence	Coherence	SCT Stage
Relational maturity	0.411***	–0.152**	0.204***	0.362***	0.221***	0.279***	0.357***	0.400***	0.538***
Emotional integration	0.300***	–*0.020*	0.174***	0.245***	0.274***	0.196***	0.239***	0.273***	0.647***
Value-driven action	0.372***	–0.119**	0.227***	0.288***	0.271***	0.259***	0.319***	0.350***	0.572***
Self-directed leadership	0.211***	*-0.011*	0.263***	0.117**	0.235***	0.201***	0.209***	0.239***	0.513***
Ethical alignment	0.383***	–0.145**	0.116**	0.299***	0.252***	0.225***	0.348***	0.374***	0.536***
Holistic thinking	0.291***	–*0.041*	0.203***	0.224***	0.469***	0.162**	0.283***	0.274***	0.723***
Societal and cultural awareness	0.138**	*0.013*	0.141**	0.115**	0.323***	*0.073*	*0.087*	0.098**	0.541***
Self-transcendent awareness	0.369***	–*0.093*	0.253***	0.273***	0.328***	0.243***	0.334***	0.376***	0.645***
Language complexity	0.170***	*0.022*	0.169***	0.118**	0.386***	0.136**	0.143**	0.140**	0.742***

Analytic *N* = 444 (see Section 2.1.3). **q < 0.05, ***q < 0.001, where q-values are Benjamini–Hochberg FDR-adjusted over all 81 tests.

Italicized correlations are non-significant after FDR correction (q ≥ 0.05). **Bold** marks moderate-to-strong effects, defined as |ρ| ≥ 0.35.

Specific patterns further support construct validity. *Relational maturity* showed the strongest personality associations, with moderate positive correlations with Agreeableness (ρ = 0.411) and Actor Coherence (ρ = 0.400), alongside elevated Value-Behavior Coherence (ρ = 0.357) and a strong association with WUSCT stage (ρ = 0.538). *Emotional integration* was most closely associated with Narrative Thematic Coherence (ρ = 0.287)—the integration of recurring emotional themes across self-defining memories—and was unrelated to hedonic wellbeing (SPANE Positive/Negative, Satisfaction with Life; all |ρ| < 0.04, ns), consistent with this indicator capturing emotional maturity rather than positive mood; in Dataset 7 it correlated ρ = 0.47 with the MSCEIT, a performance-based emotional reasoning test. Indicators reflecting cognitive complexity differentiated as expected: *Holistic thinking* related modestly to personality (Agreeableness ρ = 0.291; Neuroticism ns), but showed the strongest link to the Author Composite (ρ = 0.469) and a very strong association with WUSCT (ρ = 0.723), consistent with this indicator capturing integrative synthesis rather than interpersonal warmth. *Self-directed leadership* showed smaller but uniformly significant associations across constructs (Agreeableness ρ = 0.211; IAF ρ = 0.201; WUSCT ρ = 0.513); in a supplementary analysis using Dataset 2, it also correlated with occupational seniority (ρ = 0.342, *p* = 0.005). *Societal and cultural awareness* showed weaker personality and coherence links than other indicators, but maintained moderate associations with the Author Composite (ρ = 0.323) and WUSCT (ρ = 0.541), consistent with this indicator capturing verbal-conceptual sophistication; supplementary analysis confirmed a significant association with educational attainment in Dataset 2 (ρ = 0.188, *p* < 0.001). *Self-transcendent awareness* showed the largest known-groups separation of any indicator: contemplative practitioners in Dataset 7 scored substantially higher than matched controls (Hedges' *g* = 1.43, *p* < 0.001; Madl and Lazar, in preparation). More broadly, WUSCT correlations were uniformly large for the socio-emotional and principled indicators (ρ ≈ 0.51–0.72), underscoring alignment with developmental stage.

Taken together, this pattern of theoretically coherent correlations indicates that the AI indicators capture variance related to established markers of personality integration, coherence, and mature functioning, in ways consistent with their conceptual definitions (see Supplementary Appendix A for indicator details).

Next, to test whether the AI maturity indicators capture unique variance beyond broad personality dimensions, we conducted hierarchical multiple regression (HMR). For each external variable (including the expert-rated WUSCT stage in the Fournier et al. dataset), outcomes were first predicted from the five Big Five traits (Model 1), then from Model 1 plus the set of AI-derived indicators (Model 2). Table 4 reports the incremental variance explained (Δ*R*^2^) by the AI indicators. Trait outcomes are included in Table 4 not as incremental-validity tests in the usual sense—predicting one Big Five trait from the remaining four traits plus the indicators partly restates the zero-order structure of Table 3—but as a convergent/discriminant pattern check: maturity indicators should add variance for maturity-adjacent traits (Agreeableness, Conscientiousness, and Openness) and should not for Neuroticism and Extraversion. The substantive incremental-validity claims rest on the non-trait outcomes (coherence indices, autonomous functioning, and expert-rated stage).

**Table 4:** Incremental validity of AI maturity indicators beyond big five traits (hierarchical multiple regression).

Dependent variable	R^2^ model 1	R^2^ model 2	ΔR^2^	*F* change	*p*-value (Δ*R*^2^)
	(Big Five)	(Big Five + AI Indic.)	(AI Indic.)		
Big Five Agreeableness	0.264	0.408	0.143	10.867	<0.001***
Big Five Neuroticism	0.385	0.400	0.015	1.095	0.365
Big Five Extraversion	0.298	0.325	0.026	1.751	0.076
Big Five Conscientiousness	0.237	0.301	0.063	4.065	<0.001***
Big Five Openness	0.154	0.221	0.067	3.877	<0.001***
IPIP Unassuming-Ingenuous	0.518	0.532	0.014	1.304	0.232
Author Composite	0.064	0.254	0.190	11.421	<0.001***
Autonomous Functioning Index (IAF)	0.213	0.246	0.033	1.958	0.043*
Value-Behavior Coherence	0.263	0.310	0.048	3.080	0.001***
Actor Coherence	0.491	0.521	0.030	2.797	0.003**
WUSCT Stage (Expert Rated)	0.166	0.757	0.591	109.041	<0.001***

Analytic *N* = 444. Model 1 predictors = Big Five traits; for trait outcomes, the outcome trait was excluded from Model 1.

Model 2 predictors = Big Five + 9 AI Maturity Indicators (8 LLM-derived + Language Complexity). ΔR^2^ indicates change due to adding AI indicators.

^*^p < 0.05; ^**^p < 0.01; ^***^p < 0.001.

Results show clear incremental validity. The AI indicators added substantial variance beyond the Big Five for markers of personality integration, especially the Author Composite (Δ*R*^2^ = 0.190, *p* < 0.001), Value-Behavior Coherence (Δ*R*^2^ = 0.048, *p* = 0.001), and Actor Coherence (Δ*R*^2^ = 0.030, *p* = 0.003). When treating Big Five domains as outcomes, significant increments emerged for Agreeableness (Δ*R*^2^ = 0.143, *p* < 0.001), Conscientiousness (Δ*R*^2^ = 0.063, *p* < 0.001), and Openness (Δ*R*^2^ = 0.067, *p* < 0.001)—the three traits most conceptually linked to developmental maturity—suggesting that the indicators capture maturity-relevant variance beyond broad trait structure. Increments for Neuroticism (Δ*R*^2^ = 0.015, *p* = 0.365), Extraversion (Δ*R*^2^ = 0.026, *p* = 0.076), and IPIP Unassuming-Ingenuous (Δ*R*^2^ = 0.014, *p* = 0.232) were not significant, consistent with the indicators reflecting developmental maturity rather than emotional adjustment or temperament. A small but statistically significant increment emerged for Autonomous Functioning (IAF; Δ*R*^2^ = 0.033, *p* = 0.043).

The largest increment emerged for expert-rated WUSCT scores (Δ*R*^2^ = 0.591, *p* < 0.001), where adding the indicators increased total *R*^2^ from 16.6% (Big Five only) to 75.7%. The indicators thus capture the core construct of ego development, as judged by human experts, far more effectively than broad personality traits alone.

Taken together, these analyses on an independent dataset support the construct validity of the AI-derived maturity indicators. The indicators correlate with related constructs assessing personality style, coherence, and autonomy in theoretically expected patterns, and demonstrate substantial incremental validity in predicting these outcomes, as well as expert EDT ratings, beyond the Big Five traits. This is consistent with the indicators capturing developmental variance central to Ego Development Theory.

### 3.5 Validation of the language complexity indicator against integrative complexity

We evaluated the ability of the Language Complexity (LC) indicator to predict held-out human-rated Integrative Complexity (IC) scores on two distinct corpora, using 10-fold cross-validation (see Section 2).

First, on Dataset 5 (the UBC/Suedfeld expert-anchored reference set for Integrative Complexity), Language Complexity predictions on held-out data achieved a high intraclass correlation from a two-way random-effects model: ICC(2,1) = 0.771, 95% CI [0.700, 0.830] (Krippendorff's α = 0.770, reported for comparison with Conway et al., 2014).

Second, this performance replicated on the large-scale Jakob et al. (2023) corpus of naturalistic online discourse (Dataset 6). Despite the noisier context of social media comments, the LC scores on held-out data maintained similar reliability against trained raters: ICC(2,1) = 0.727, 95% CI [0.710, 0.750] (Krippendorff's α = 0.727).

This level of performance (α and ICC both in the range 0.73–0.77) exceeds the previously reported state-of-the-art for dedicated automated IC scoring systems in our samples (α = 0.62; Conway et al., 2014). The consistent performance across both didactic exemplars and noisy, real-world text suggests that the evolutionarily discovered LC features generalize across writing contexts as measures of cognitive structural complexity.

Language Complexity was strongly associated with stage (Spearman ρ = 0.879, 95% CI [0.861, 0.890] in Dataset 2; ρ = 0.742, 95% CI [0.688, 0.788] in Dataset 3, consistent with Table 3), and remained strongly associated with stage after accounting for verbosity: partial Spearman ρ = 0.560, 95% CI [0.500, 0.613], *n* = 851 (controlling for log words). In a proportional-odds model including log words, LC showed an OR per 1 SD of 27.36 (95% CI [16.35, 45.80]); LR $\chi_{\left(1\right)}^{2}=203.45$, *p* = 3.70 × 10^−46^. Controlling instead for Flesch–Kincaid grade yielded partial Spearman ρ = 0.732 (95% CI [0.694, 0.765]) and an OR per 1 SD of 27.39 (95% CI [18.88, 39.73]); LR $\chi_{\left(1\right)}^{2}=498.03$, *p* = 2.55 × 10^−110^, *n* = 851.

These results indicate that the Language Complexity indicator captures variance in EDT stage that is not attributable to text length or readability. Complexity alone, however, was not sufficient to predict EDT stage: a classifier using only Language Complexity and omitting all other maturity indicators attained 63.2% accuracy (95% CI [59.9, 66.4]; κ = 0.766, 95% CI [0.734, 0.793]).

## 4 Discussion

The central finding of this study is that an interpretable, language-model-based scoring pipeline reproduced expert ego development ratings from the Sentence Completion Test (SCT) on held-out data and in a blinded replication with an independently trained scorer, with error profiles in which most misclassifications fall on adjacent stages. Our primary goal was to develop a scalable alternative to manual SCT scoring that makes developmental assessment accessible to a broader research and practitioner audience, while surfacing interpretable, theory-aligned indicators of maturity that emerge from data rather than being predetermined by scoring manuals. Robust assessment of adult development matters because later stages are linked empirically to capacities relevant to individual well-being and societal challenges, including complex thought, navigating uncertainty, empathy, and effective leadership (Joiner, 2011; Binder, 2023; Bushe and Marshak, 2016; Manners and Durkin, 2001; Torbert and Livne-Tarandach, 2009; Boiral et al., 2014).

Beyond prediction, the approach yields a compact set of interpretable maturity indicators (e.g., Language Complexity) that show meaningful construct validity, remain predictive after controlling for verbosity, and organize in ways congruent with Ego Development Theory (EDT). The evidence is strongest for measurement automation; evidence that the indicators constitute dimensions of developmental structure is more preliminary (Section 4.1). Taken together, these results are consistent with a multidimensional view of maturity and open avenues for longitudinal and population-scale research.

### 4.1 Prediction vs. discovery: circularity and criterion dependence

Before interpreting the discovered indicators, an epistemological concern must be addressed directly. A model whose optimization target is expert-assigned scores cannot, by that fact alone, be credited with discovering the latent structure of development. At best, such a model recovers the structure of ego development *as operationalized by expert scoring*; at worst, it reconstructs the statistical geometry of a particular scorer's behavior—stylistic regularities, semantic proxies, or manual-specific conventions—without capturing anything psychologically deeper. Because our pipeline was trained on expert-scored data and fine-tuned on scoring-related literature, this concern applies with full force to the accuracy results of Section 3.1 taken in isolation.

Four classes of evidence constrain this deflationary interpretation. First, *direct tests of shallow mechanisms*: the most obvious superficial routes to reproducing expert scores were tested and found insufficient. Classifiers built on lexical content alone (bag-of-words, 35.8%) or on state-of-the-art semantic embeddings (36.7%) fell far short of expert agreement, as did frontier models prompted with the full scoring manual (40.1%–44.9%); and Language Complexity remained strongly associated with stage after controlling for verbosity and readability, with a complexity-only classifier reaching just 63.2% (Section 3). Whatever regularities the model exploits, they are not reducible to response length, readability, surface word choice, or the manual text itself. Second, *cross-tradition transfer*: the indicators were evolved solely against item-level scores from the Loevinger-tradition composite corpus (Dataset 1), which aggregates protocols scored across multiple laboratories and decades, and were frozen before any contact with other data. The same frozen indicators nevertheless supported 86.1% agreement with Cook-Greuter's protocol-level ratings (Dataset 2) and, beyond the Big Five traits, explained substantial additional variance in WUSCT stage scored by a third, unrelated group of raters (Δ*R*^2^ = 0.591, for a total *R*^2^ of 0.757; Dataset 3). Conventions idiosyncratic to a single scorer or manual cannot account for transfer across three criterion sources spanning two scoring traditions. Third, *blinded replication*: agreement of 85.1% with an independently trained scorer under mutual blinding (Dataset 7) shows that the model's outputs converge with expert judgment beyond the criterion scorer it was calibrated to—though this replication remains within the Cook-Greuter scoring tradition; the Loevinger-scored discovery and validation corpora (Datasets 1 and 3) extend the evidence across the two related scoring systems, which nonetheless share a common theoretical lineage. Fourth, and most importantly, *external criteria that no scorer produced and the optimization never saw*: the indicators showed theoretically differentiated associations with performance-based emotional reasoning (Emotional Integration–MSCEIT ρ = 0.47), narrative coherence, autonomous functioning, and—in non-SCT text—behavioral outcomes such as charitable donation (Value-Driven Action), negotiation performance (Emotional Integration), and group problem-solving and legislative effectiveness (Holistic Thinking). Equally informative are the discriminant nulls: the indicators added no significant variance for Neuroticism or Extraversion, and Emotional Integration was unrelated to hedonic well-being. A model that had merely internalized scorer-specific conventions would have no evident reason to produce this differentiated, theory-congruent pattern of convergent and discriminant relations with criteria outside the scoring ecosystem.

These considerations constrain, but do not eliminate, the circularity concern. The stage criterion itself remains anchored to human expert judgment—no scorer-independent gold standard for ego development exists—so all accuracy claims are claims about convergence with a scoring tradition, not accuracy against an external ground truth. Several external criteria are themselves text-derived and may share method variance with the indicators. While the direct tests above exclude the crudest shallow mechanisms, subtler proxies—scorer-specific responses to particular constructions, or demographic correlates such as education-linked eloquence—cannot be fully ruled out. And the discovery, evaluation, and validation samples all consist predominantly of educated, Western adults, so shared population characteristics may inflate apparent generality. We therefore frame the indicators as *candidate dimensions with demonstrated criterion and construct validity*, not as established components of a latent developmental structure. Adjudicating the stronger claim will require designs that break the dependence on concurrent expert scoring: multimethod measurement models with invariance testing, longitudinal designs tracking within-person change, and preregistered tests of novel predictions derived from the indicators.

### 4.2 A multidimensional view of maturity

A recurring psychometric challenge for EDT is that SCT responses seldom behave as if generated by a single latent trait, a pattern sometimes framed as a measurement flaw (Thornton, 2023). In practice, models that impose unidimensionality with local independence (e.g., Rasch/1PL) can misfit when the construct is high-dimensional; violations of these assumptions need not imply invalidity of EDT but may simply call for models that accommodate multiple capacities (e.g., Multidimensional Item Response Theory, bifactor, or network approaches—McDonald, 2000; Marsman et al., 2018; DeMars, 2006).

Our results are more consistent with adult development unfolding within a space spanned by partially independent capacities than with a single continuum. The pipeline yielded interpretable indicators spanning socio-emotional, cognitive, transpersonal, and linguistic domains. These indicators explained non-redundant variance in expert ratings after mutual adjustment, and showed external validity against independent criteria such as narrative coherence, autonomous functioning, and integrative complexity (Table 4). As expected for indicators selected for association with a common developmental criterion, the indicator space shows a strong positive manifold: an eigendecomposition of the inter-indicator correlations (Supplementary Appendix B) yields a dominant general dimension, with a second component—contrasting cognitive-linguistic with socio-emotional capacities—at the conventional retention boundary. This structure parallels other domains organized around a general factor (e.g., cognitive abilities), in which meaningful differentiation nonetheless exists below the apex. Consistent with this, the indicators show functional differentiation: substantial accuracy loss when the model is reduced to Language Complexity alone (63.2% vs. 86.1%), non-redundant contributions after mutual adjustment, and theoretically differentiated external correlates.

Although EDT identifies stage-structured configurations of such capacities, it does not posit a simple linear scale. Rather, later development involves differentiation followed by integration (e.g., a gradual loosening and re-integration of self boundaries; Cook-Greuter, 1999; Cook-Greuter and Soulen, 2007). Consistent with this view, stages occupy largely contiguous, ordered regions of the projected feature space (Figure 2), though, as noted in Section 3.3, these projections are descriptive rather than confirmatory. Furthermore, the confusion matrix (Figure 1) shows that misclassifications are overwhelmingly confined to immediately adjacent stages. Given the limitations of our data, we do not assert a definitive factor structure; instead, we claim that the joint, interpretable, and incrementally predictive indicators are consistent with a multidimensional account of maturity.

### 4.3 Complexity is necessary but not sufficient

A related critique holds that sentence completion tasks may reward verbosity rather than structural maturity (Manners and Durkin, 2001; Lanning et al., 2018). Our results indicate that Language Complexity (our linguistically grounded proxy for integrative complexity) remains predictive after controlling for verbosity and readability, and its associations extend beyond stage to independent benchmarks (e.g., integrative complexity, narrative coherence).

At the same time, complexity alone does not exhaust what the model learns: reducing the architecture to this single aspect of maturity lowers predictive accuracy from 86.1% to 63.2%. Socio-emotional and ethical indicators retain incremental predictive value and show differential external links (e.g., with agreeableness, autonomous functioning, and coherence composites). Thus, hierarchical complexity appears *necessary* for higher-stage responses but *not sufficient* by itself; more complex, more mature language is typically accompanied by shifts in relational stance, value alignment, and perspective taking captured by the other indicators.

### 4.4 Broader implications for personality science

#### 4.4.1 Implications for developmental theory

By recovering interpretable indicators that jointly predict stage and by showing predominantly adjacent-stage errors, our results are consistent with stage-structured development that unfolds across partially independent capacities rather than along a single latent axis. Preliminary evidence from the Pennebaker analysis is consistent with the theoretical expectation of alternating differentiation and integration phases, though this finding rests on indirect proxies and AI-predicted stages and requires confirmation with expert-rated longitudinal data. By linking the AI-derived indicators to external measures like narrative coherence, autonomous functioning, and integrative complexity, these findings add external correlates consistent with the theory. Our results also suggest that higher-stage language reflects broader shifts in appraisal and value-coordination, not merely response length or stylistic variance. We view these patterns as convergent evidence for a structured, multidimensional account of adult development, while recognizing that definitive causal or factor-structural claims will require dedicated designs (e.g., multimethod, longitudinal measurement models with invariance tests).

#### 4.4.2 Implications for psychological method

Automated, interpretable scoring enables designs that were previously impractical: high-throughput longitudinal studies, quasi-experiments (e.g., before/after intensive trainings), and population-representative sampling with sufficient power to model subgroup differences and developmental trajectories. Because the indicators are human-interpretable, they can serve as theory probes—supporting targeted hypothesis tests (e.g., whether socio-emotional stance mediates links between complexity and outcomes) and informing item or prompt refinement. The evolutionary feature-discovery framework is portable: analogous pipelines could operationalize adjacent constructs (e.g., wisdom-related reasoning, moral perspective taking) while preserving interpretability and auditability.

Although the model was developed and validated on SCT responses, preliminary evidence suggests the maturity indicators generalize to other open-ended text. The Pennebaker analysis (Dataset 4) showed that AI-predicted stages from stream-of-consciousness essays reproduced theoretically expected alternating patterns. Across five additional corpora spanning diverse genres, individual indicators predicted domain-appropriate behavioral outcomes beyond personality traits: Value-Driven Action predicted charitable donation (ρ = 0.193, surviving Big Five and moral-foundations controls), Holistic Thinking predicted legislative effectiveness, persuasive success (full sample *d* = 0.166, *N* ≈ 2, 900 pairs; *d* = 0.112 in the word-count-matched subset, *N* ≈ 800, *p* = 0.001), and group problem-solving accuracy, and Emotional Integration predicted negotiation outcomes. Effect sizes (ρ ≈ 0.10–0.22) are comparable to Big Five personality–performance associations derived from validated questionnaires (Barrick and Mount, 1991; Zell and Lesick, 2022), and exceed typical automated integrative complexity effects (Houck et al., 2014; Grüning and Apitz, 2024). We present these analyses explicitly as exploratory and hypothesis-generating; they await pre-registered replication. Full dataset descriptions and analyses are reported in Supplementary Appendix D.

#### 4.4.3 Practical and ethical considerations

The validation evidence presented here concerns automated *stage estimation*; the validity and effects of downstream applications such as developmental feedback or coaching reports are distinct questions requiring separate evaluation. Given potential applied interest (e.g., leadership development, coaching), careful governance is essential. Recommended safeguards include: (i) use in research and low-stakes formative contexts with clear communication of uncertainty; (ii) human-in-the-loop review for any consequential decisions; (iii) routine subgroup performance audits and length-robustness checks; and (iv) privacy-preserving workflows. Careful framing and communication of uncertainty is particularly important to prevent category errors (treating probabilistic stage estimates as fixed traits).

### 4.5 Limitations

Despite the promising results, several limitations should be acknowledged.

The primary accuracy criterion (Dataset 2) was produced by a single expert scorer—Cook-Greuter, who is also an author of this paper—and the classifier is therefore calibrated to one scoring tradition; scorer-specific idiosyncrasies cannot be fully excluded. The blinded replication with an independently trained scorer yielded near-identical agreement (85.1%), and the indicators also predicted stage as scored by a third, unrelated group of raters (Dataset 3), but further testing across a wider range of scorers—ideally against consensus-scored protocols—remains desirable. More fundamentally, because no scorer-independent criterion for ego development exists, high agreement demonstrates convergence with expert judgment rather than accuracy against an external ground truth (see Section 4.1). The model is currently validated only for English-language responses. The model relies on a fine-tuned Qwen2.5 LLM, and potential limitations or biases inherent in the base architecture may persist. In addition, the evolutionary discovery procedure is highly flexible; despite the safeguards described in Section 2.3, we cannot rule out that some discovered features exploit corpus-specific regularities of the discovery dataset. Relatedly, both the discovery and evaluation corpora are dominated by conventional-range observations (86% of Dataset 1 item ratings fall at E4–E6, and 67% of Dataset 2 protocols at Stages 4 and 4/5); the evolutionary fitness signal was therefore driven primarily by mid-range discriminations, and the discovered indicators may be less well-calibrated at the developmental extremes—consistent with the lower recall observed for Stage 5/6. The source datasets feature relatively specific populations (e.g., individuals with higher education, predominantly midlife adults), and validation across more diverse demographic groups remains an important direction for future work. The primary analyses are cross-sectional, limiting causal inferences about developmental processes over time. Finally, the analyses applying the model beyond standard SCT protocols—stage prediction from stream-of-consciousness essays (Dataset 4) and maturity indicator scoring of non-SCT text across five additional corpora (Supplementary Appendix D)—were exploratory. However, the convergence of consistent effects across six independent datasets, diverse text genres, and multiple behavioral outcomes lends initial plausibility to cross-genre generalization; pre-registered replication is needed to confirm these findings.

### 4.6 Conclusion

We introduced an evolutionary, interpretable approach that scores SCT responses with agreement comparable to trained raters, while yielding indicators consistent with a multidimensional account of ego development. Beyond strong predictive performance and predominantly adjacent-stage errors, the indicators—especially language complexity alongside socio-emotional and ethical cues—show external validity and remain incrementally informative after controlling for verbosity. These results suggest that later-stage responses reflect coordinated changes across partially independent capacities rather than movement along a single latent axis.

The measurement claim is now supported by convergent evidence across three criterion sources and an independently verified blinded replication; claims concerning the structure of development remain provisional and invite targeted tests. Priority next steps include validation across further scorers and traditions; length-robust and multilingual extensions with invariance checks; and longitudinal designs that track within-person change and probe mechanisms (e.g., how shifts in socio-emotional stance mediate links between complexity and outcomes). By pairing interpretable indicators with verifiable evaluation artifacts, this work makes scalable, theory-driven developmental assessment more feasible. Used with appropriate safeguards and human oversight, such tools can broaden access to developmental measurement while advancing cumulative science on adult maturity.

## Data Availability

This research did not collect new data. The primary analyses relied on seven pre-existing archival datasets (Datasets 1–7); five additional public corpora were used in the exploratory analyses of Supplementary Appendix D, with sources cited there. Access to the original raw data remains at the discretion of the original data custodians (Dataset 1 Lanning et al. (2018), Dataset 2 Cook-Greuter (1999), Dataset 3 Fournier et al. (2022), Dataset 4 Pennebaker and King (1999), Dataset 5 Conway et al. (2014), Dataset 6 Jakob et al. (2023), Dataset 7 Singleton et al. (2021)). To enable independent verification, we provide essential analysis materials in the Supplementary material and via an anonymous, view-only Open Science Framework (OSF) repository accessible here. Upon acceptance, a permanent public OSF release with a DOI will be issued. The repository includes: (i) *predictions.csv*^1^ with de-identified model outputs keyed to anonymized participant IDs, *fold_assignments.csv*^2^ with participant-level cross-validation splits, *ablation_direct_predictions.csv*^3^ with the direct-scoring stage predictions underlying the ablation reported in Section 3.1, and *eval_and_plot.py*^4^ to reproduce reported accuracy metrics and figures on Dataset 2, (ii) *baseline_score_with_manual.py*^5^ with code to reproduce the OpenAI, embedding-based, and bag-of-words scoring baseline accuracies, (iii) *fournierpdfX_final.feather*^6^ and *fournier2022analysis.py*^7^ to reproduce external construct and incremental validity tables and results based on Dataset 3, (iv) *essays_data.feather*^8^ and *pennebakeranalysis.py*^9^ to reproduce results based on Dataset 4, (v) *dataset_5_results.csv*^10^, *dataset_6_results.csv*^11^, and *m_ic_analysis.py*^12^ to reproduce results based on Datasets 5 and 6, (vi) *model_card.pdf* ^13^. The SHA-256 hashes in the footnotes provide tamper-evident, version-exact identifiers for all files, enabling reproducibility. All LLM inferences were run deterministically (temperature=0, top-p=1); all random seeds were set to 42. A frozen inference artifact for verification is identified by digest: SHA-256 b569fba22adf1c8bcfdf263eba49ed1b411c26db ec0332013c575902e5a2eace, and accessible through API upon request. The complete evolved rubric set (75 sub-indicators) is likewise identified by digest SHA-256 670cdcbdfe99 870bdcedc76a63ae47ce2a4a7154603bae5177f45fe6aad07bc0, permitting confidential audit of bundle identity without public disclosure. All released files are de-identified and contain no PII. The released materials support independent *verification* of all reported results: metrics and figures can be regenerated exactly from the released predictions, and model outputs can be verified against the frozen inference artifact. Full *reproduction* of the discovery methodology would additionally require the complete prompt set and model weights, which licensing constraints prevent us from releasing; an example evolved rubric is provided in Section 2.2, and a second in the Supplementary Material. For rigorous verification, peer reviewers and qualified academic researchers can access the frozen inference artifact via the API documented in the OSF model card under a standard data use agreement. A containerized binary for local execution is available upon reasonable request (requires an 80 GB-class GPU, e.g., H100 80 GB). We commit to maintaining verification access for at least 24 months post-publication.
