# Supplementary material for: Automated scoring of adult ego development from sentence completions: interpretable maturity indicators derived by evolutionary prompt optimization
Source: Front Psychol. 2026 Aug 27;17:1853871. doi: 10.3389/fpsyg.2026.1853871 (PMC13558132; doi:10.3389/fpsyg.2026.1853871)
Supplement: Supplementary file 1 [file Data_Sheet_1.pdf]

Supplementary Material

1 APPENDIX A. MATURITY INDICATORS

This appendix provides concise descriptions of the nine maturity indicators derived from the evolutionary AI approach. Empirical anchors refer primarily to correlations observed within the independent Fournier et al. (2022) dataset (Dataset 3), unless otherwise noted; supplementary anchors from Dataset 2 are indicated where available.

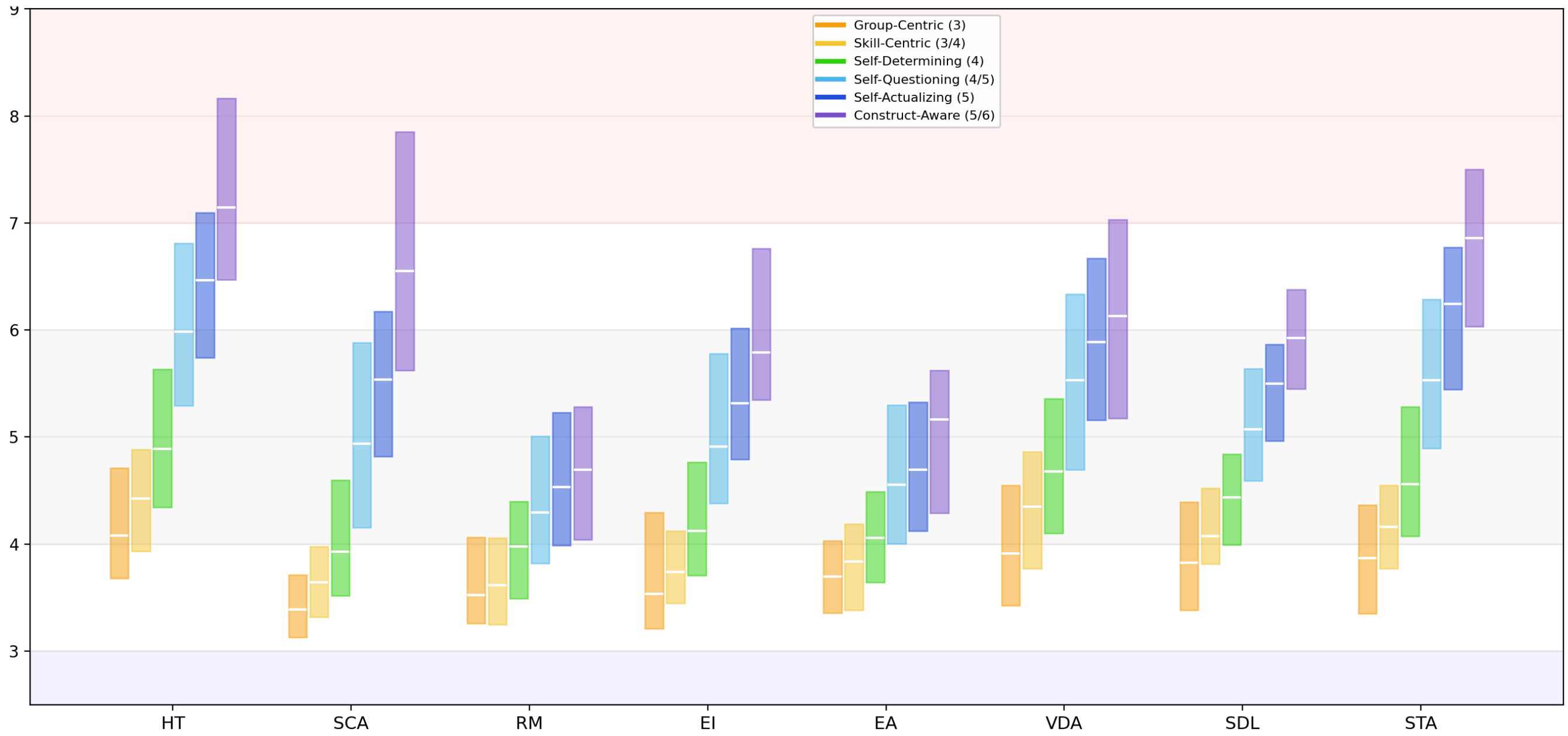

Figure S1. Maturity indicator scores by expert-assigned *item-level* stage rating (Dataset 2). Individual completions can receive item ratings outside the range of protocol-level TPRs; the figure therefore includes a Group-Centric (Stage 3) item category even though no protocol in Dataset 2 has a TPR of 3 (cf. Table 2 in the main text). Boxes show interquartile ranges; white lines indicate medians. All eight LLM-derived indicators increase monotonically across item-level stages, with the steepest differentiation occurring at different points along the developmental continuum.

1.1 Socio-Emotional and Self-Related Capacities

- **Relational Maturity**: An open orientation toward others—genuine warmth and gratitude in connection that does not depend on circumstances or require others to be alike. *Empirical anchors:* positively related to Agreeableness ($\rho = .411$) and interpersonal coherence (Actor $\rho = .400$, Value–Behavior $\rho = .357$), modestly negatively to Neuroticism ($\rho = -.152$), and strongly linked to WUSCT stage ($\rho = .538$).
- **Emotional Integration**: Navigating emotions with skill—integrating thought and feeling, reading oneself and others accurately, and receiving feedback without losing one's center. *Empirical anchors:* positive associations with Agreeableness ($\rho = .300$) and coherence (Value–Behavior $\rho = .239$, Actor $\rho = .273$), Neuroticism near zero (ns; $\rho = -.020$), and a strong relation to WUSCT ($\rho = .647$). Among the narrative coherence facets, this indicator was most closely associated with Thematic Coherence ($\rho = .287$, $p < .001$)—the ability to identify and integrate recurring emotional themes across

self-defining memories—consistent with its emphasis on integrating thought and feeling. Notably, Emotional Integration was unrelated to hedonic well-being (SPANE Positive/Negative, Satisfaction with Life; all $|\rho| < .04$, ns), consistent with this indicator capturing emotional maturity rather than positive mood. In a supplementary analysis on Dataset 7, this indicator correlated $\rho = .47$ with the MSCEIT, a performance-based measure of emotional reasoning ability.

- **Self-Directed Leadership**: Leading from inner conviction—reflecting deeply, questioning norms on their merits, and building shared purpose through who one is rather than what one enforces. *Empirical anchors:* small but uniformly significant associations with Agreeableness ($\rho = .211$), IAF ($\rho = .201$), and Author Composite ($\rho = .235$); strong with WUSCT ($\rho = .513$). In a supplementary analysis on Dataset 2, this indicator correlated with occupational seniority ($\rho = .342$, $p = .005$, up to middle management).
- **Value-Driven Action**: Acting from values rather than incentive—owning consequences fully, without deflection or excuse. *Empirical anchors:* positive links to coherence (Value–Behavior $\rho = .319$, Actor $\rho = .350$), Agreeableness ($\rho = .372$), and IAF ($\rho = .259$), small negative with Neuroticism ($\rho = -.119$), and strong with WUSCT ($\rho = .572$).

1.2 Cognitive and Principled Functioning

- **Ethical Alignment**: Grounding decisions in examined principles, widening the circle of who one considers, and giving without keeping score. *Empirical anchors:* moderate positives with Agreeableness ($\rho = .383$), IAF ($\rho = .225$), and coherence (Value–Behavior $\rho = .348$, Actor $\rho = .374$), small negative Neuroticism ($\rho = -.145$), and strong WUSCT association ($\rho = .536$).
- **Holistic Thinking**: Connecting ideas into a coherent whole—synthesizing competing or scattered perspectives into unified, structured understanding. *Empirical anchors:* modest ties to personality (Agreeableness $\rho = .291$; Neuroticism ns), the strongest link to narrative coherence of any indicator (Author Composite $\rho = .469$), and a very strong WUSCT association ($\rho = .723$).
- **Societal & Cultural Awareness**: Reading the cultural, structural, and intellectual currents that shape how people see—and engaging diverse traditions with genuine appreciation. *Empirical anchors:* moderate Author Composite ($\rho = .323$) and WUSCT ($\rho = .541$); weaker personality and coherence links, consistent with this indicator capturing verbal-conceptual sophistication rather than interpersonal style. In a supplementary analysis on Dataset 2, this indicator correlated with educational attainment ($\rho = .188$, $p < .001$).

1.3 Transpersonal Development

- **Self-Transcendent Awareness**: Witnessing one's experience rather than being fused to it, and stepping back from what may have hold—reward, reputation, certainty, self-image—so one can act from something deeper. *Empirical anchors:* positive with Agreeableness ($\rho = .369$), IAF ($\rho = .243$), and coherence (Value–Behavior $\rho = .334$, Actor $\rho = .376$); Neuroticism near zero (ns; $\rho = -.093$); strong with WUSCT ($\rho = .645$). In the Dataset 7 neuroimaging cohort, contemplative practitioners scored substantially higher than matched controls (Hedges' $g = 1.43$, 95% CI [0.83, 2.03], $p < .001$; $n = 45$)—roughly two and a half times the average effect of meditation on psychological outcome measures in the most comprehensive meta-analysis available (mean r = .28, corresponding to $d \approx 0.57$; Sedlmeier et al., 2012, *Psychological Bulletin*). This indicator was strongly associated with adaptive self-reflection ($r = .49$, $p < .001$) but unrelated to rumination ($r = .01$, ns), consistent with capturing integrative self-examination rather than repetitive negative self-focus (Madl & Lazar, in preparation).

1.4 Linguistic Dimension

- **Language Complexity**: Constructing precise, layered language that carries nuance—the architecture of expression, not just vocabulary. Scored algorithmically from evolutionarily-discovered linguistic features rather than LLM-rated rubrics. *Empirical anchors:* Very strong correlation with Integrative Complexity (ICC(2,1) $\approx .73 - .77$) and with developmental stage (WUSCT $\rho = .742$), moderate relation to narrative coherence (Author Composite $\rho = .386$), and comparatively weaker links to Agreeableness ($\rho = .170$) and IAF ($\rho = .136$).

2 APPENDIX B. INTER-INDICATOR CORRELATIONS

Table S1 presents the Spearman rank-order correlations among the nine maturity indicators, computed on Dataset 3 ($N = 444$; two participants with null [missing] sentence-completion responses in the source data were excluded).

Table S1. Inter-indicator correlations (Spearman's ρ, Dataset 3, N=444). RM=Relational Maturity, EI=Emotional Integration, VDA=Value-Driven Action, SDL=Self-Directed Leadership, EA=Ethical Alignment, HT=Holistic Thinking, SCA=Societal & Cultural Awareness, STA=Self-Transcendent Awareness, LC=Language Complexity.

	RM	EI	VDA	SDL	EA	HT	SCA	STA	LC
RM	—								
EI	.55	—							
VDA	.77	.61	—						
SDL	.53	.58	.69	—					
EA	.69	.60	.74	.60	—				
HT	.57	.54	.55	.47	.57	—			
SCA	.31	.40	.32	.31	.31	.60	—		
STA	.68	.66	.87	.70	.72	.58	.33	—	
LC	.46	.56	.48	.48	.49	.69	.60	.49	—

The indicators share a common positive manifold (all $\rho > 0$; median off-diagonal $\rho = .57$), consistent with a general maturity factor that runs through them. Within this structure, two clusters are apparent. The socio-emotional and principled indicators (RM, VDA, EA, STA) intercorrelate most strongly ($\rho \approx .68$–.87), with VDA and STA showing the highest association ($\rho = .87$)—both capture value-aligned orientation, though at different levels of abstraction (behavioral enactment versus meta-aware witnessing). A second, more cognitive-linguistic cluster links HT, SCA, and LC ($\rho \approx .60$–.69), with weaker ties to the socio-emotional group. This separation reinforces the finding from the main text that linguistic complexity alone is insufficient for stage prediction: the socio-emotional indicators capture developmental variance that is partially independent of cognitive-linguistic sophistication.

To characterize the effective dimensionality of the indicator space, we performed an eigendecomposition of the correlation matrix in Table S1. The eigenvalues were 5.53, 1.18, 0.54, 0.43, 0.37, 0.30, 0.29, 0.26, and 0.11; the first two components account for 61.4% and 13.1% of the variance, respectively. The second component—contrasting the cognitive-linguistic cluster (HT, SCA, LC) with the socio-emotional cluster—lies at the conventional retention boundary: it is retained under the Kaiser criterion ($\lambda > 1$) and falls marginally below the 95th-percentile threshold of Horn's parallel analysis at $N = 444$ (1.18 vs. 1.19). A dominant general dimension is expected here by construction: each indicator was selected for its association with the same developmental criterion, which induces positive intercorrelations among the indicators (a positive manifold, analogous to that observed among cognitive abilities); shared method variance (a single LLM scores all eight rubric-based indicators) contributes further. The main text's

multidimensionality claims accordingly rest on functional differentiation—incremental prediction and theoretically differentiated external correlates—in addition to this factorial structure. We note that this analysis characterizes the nine-indicator interpretive layer; the classifier itself operates on the underlying 75-dimensional sub-indicator space (Section 2.2 of the main text).

3 APPENDIX C. EVOLUTIONARY FEATURE DISCOVERY

This appendix specifies the evolutionary algorithm as implemented. Two clarifications up front. First, the adapted LLM was *frozen* throughout evolution: no retraining or weight update occurred at any point, and only the population of scoring rubrics (prompts) evolved. Second, the genetic operators (mutation and crossover) were performed by the LLM itself through few-shot meta-prompts, following the paradigm of LLM-driven prompt evolution over a fixed model (EvoPrompt; Guo et al., 2024), rather than through hard-coded string operations. An earlier version of this appendix reproduced pseudocode adapted from the EvoPrompting framework (Chen et al., 2023), which evolves model architectures and includes per-generation retraining; that pseudocode did not describe the present implementation and has been replaced by the specification below.

Algorithm (as implemented).

1. **Initialization.** Create an initial population of candidate scoring rubrics, seeded from Loevinger's four developmental domains (character, interpersonal style, conscious preoccupations, cognitive style).
2. **Scoring.** For each candidate rubric, the frozen LLM rates a fixed sample of Dataset 1 completions on the rubric's 1–10 scale.
3. **Fitness evaluation.** The fitness of the current rubric set is the cross-validated accuracy of a multinomial logistic regression predicting expert item-level stage ratings from the rubric outputs.
4. **Pruning.** Remove rubrics not significantly associated with item-level stage, and rubrics whose removal does not reduce fitness (conditional redundancy).
5. **Variation.** Generate offspring rubrics from the surviving rubrics via LLM meta-prompts that paraphrase, specialize, combine, or contrast them (implicit mutation and crossover).
6. **Termination.** Repeat from Step 2 until fitness improves by less than 1% over 10 consecutive generations.

A reverse-keyed example rubric. In addition to the example rubric in Section 2.2 of the main text, the following illustrates a reverse-keyed rubric, demonstrating that evolved rubrics are not uniformly positively keyed (all rubrics are rated 1–10; reverse-keyed scores are recoded as $11 - x$ before aggregation):

> **Black-and-White Thinking:** The tendency of the sentence completion to view issues and concepts in simplistic, either/or terms.
> 1–3: Nuanced perspective acknowledging complexity and multiple viewpoints.
> 4–6: Some recognition of nuance, but still tends towards simplification.
> 7–10: Extreme polarization, absolute statements, and oversimplified dichotomies.

4 APPENDIX D. GENERALIZATION TO NON-SCT TEXT

To assess whether the maturity indicators capture developmental signal beyond Sentence Completion Test responses, we applied the same LLM used for SCT scoring to five publicly available datasets spanning diverse text genres and behavioral outcomes. All text was scored at the passage level; participants with

fewer than 40 words of text were excluded. This appendix describes the datasets, scoring procedures, and results summarized in the main text.

4.1 D.1. Persuasion for Good (P4G)

Dataset. The Persuasion for Good corpus (Wang et al., 2019) contains 1,017 dialogues in which Amazon Mechanical Turk workers attempted to persuade a partner to donate to Save the Children. Participants completed Big Five personality inventories, moral foundations questionnaires, and Schwartz value surveys. The outcome variable was actual donation amount. We scored $N = 2{,}026$ participants (both persuaders and persuadees) with ≥ 40 words of dialogue.

Scoring. All messages from each participant were concatenated into a single passage and scored once on Holistic Thinking (HT), Emotional Integration (EI), and Value-Driven Action (VDA).

Results. Persuadee VDA scores predicted charitable donation ($\rho = .193$, $p < .0001$). This effect survived controls for Big Five personality ($p < .0001$) and for Big Five personality plus moral foundations jointly ($\beta = .033$, $p = .004$). Persuadees in the top VDA quartile donated at a rate of 66%, compared with 40% in the bottom quartile (Mann-Whitney $p < .0001$). Additionally, persuader HT predicted whether the partner donated (logistic OR $= 1.25$, $p = .010$, controlling for Big Five).

4.2 D.2. Winning Arguments (ChangeMyView)

Dataset. The Winning Arguments corpus (Tan et al., 2016) contains 4,263 matched pairs from Reddit's r/ChangeMyView forum. Each pair consists of a successful argument (awarded a delta by the original poster, indicating their view changed) and an unsuccessful argument addressing the same post. This matched design controls for topic and original poster, isolating the quality of argumentation. We scored $N = 2{,}878$ pairs in which both arguments contained ≥ 100 words.

Scoring. Each argument (a single Reddit comment) was scored as-is on HT. For comparison, all texts were also scored for integrative complexity using a linguistic feature regressor and GPT-4.1-mini.

Results. Successful arguments scored higher on HT than their matched unsuccessful counterparts ($d = 0.166$, $p < .000001$). Critically, after restricting to word-count-matched pairs ($|\Delta\text{words}| < 50$, $N \approx 800$), HT remained the strongest predictor ($d = 0.112$, $p = .001$), while integrative complexity measures lost significance. HT correlated modestly with word count ($\rho = .250$), whereas the linguistic complexity regressor was nearly redundant with length ($\rho = .921$), suggesting that HT captures cognitive complexity beyond verbosity.

4.3 D.3. DeliData (Group Deliberation)

Dataset. DeliData (Karadzhov et al., 2023) contains 500 group dialogues in which small groups (2–6 participants) collaborated online to solve the Wason card selection task, a well-known logical reasoning puzzle. The outcome variable was team performance: the fraction of group members who converged on the correct solution. We scored $N = 1{,}133$ participants (≥ 40 words) across 479 groups.

Scoring. All messages from each participant were concatenated and scored once on HT. Scores were then averaged to the group level.

Results. Group-level mean HT predicted team performance ($\rho = .180$, $p = .0001$). The effect survived word-count controls (OLS: HT $\beta = .008$, $p = .041$; word count independently significant). Groups in the top HT quartile achieved 76.7% performance versus 70.1% in the bottom quartile (Mann-Whitney $p < .0001$).

4.4 D.4. CaSiNo (Negotiation)

Dataset. The CaSiNo corpus (Chawla et al., 2021) contains 1,030 negotiation dialogues in which Amazon Mechanical Turk workers negotiated the allocation of camping supplies. Participants completed Big Five personality measures and Social Value Orientation assessments. The outcome variable was points scored. We scored $N = 2{,}013$ participants (≥ 40 words) on HT, EI, and VDA.

Scoring. All messages from each participant were concatenated into a single passage and scored once.

Results. EI predicted points scored beyond Big Five personality controls (OLS $\beta = .143$, $p = .003$). HT and VDA were not significant predictors of negotiation outcomes.

4.5 D.5. Congressional Speech and Legislative Effectiveness

Dataset. The Gentzkow–Shapiro–Taddy Congressional Record corpus (Gentzkow et al., 2019) contains 112,291 parsed floor speeches from the 114th Congress (2015–2016). Legislative effectiveness was measured using the Volden–Wiseman Legislative Effectiveness Scores (LES; Volden & Wiseman, 2014), which track every bill from introduction through enactment, weighted by significance. We randomly sampled $N = 200$ House legislators and scored the 5 longest speeches per legislator (≥ 40 words; 996 speeches total) on HT.

Scoring. Each speech was scored individually on HT. Scores were aggregated to the legislator level via median.

Results. HT predicted benchmark-adjusted legislative effectiveness (LES/benchmark, which controls for seniority, committee positions, and majority status; $\rho = .156$, $p = .027$). In OLS with controls for seniority, majority-party status, party affiliation, word count, and DW-NOMINATE ideology scores, HT was the strongest predictor ($\beta = .314$, $p < .001$; model $R^2 = .230$); word count and ideology were non-significant. Legislators in the top HT quartile were 70% more legislatively effective than those in the bottom quartile (mean LES 1.207 vs. 0.709). The raw correlation replicated in the 110th Congress under opposite-party majority ($\rho = .218$, $p = .002$; $N = 200$), though the controlled effect did not survive seniority adjustment in that Congress. Congressional floor speeches are often staff-prepared, though the legislator shapes argumentative framing and strategy; the results should be interpreted as reflecting legislative communication quality rather than developmental level per se.

4.6 D.6. Summary

Across five datasets, different maturity indicators predicted different behavioral outcomes in their respective theoretical domains: VDA predicted value-congruent behavior (charitable giving), HT predicted cognitive tasks (persuasion, group reasoning, legislative effectiveness), and EI predicted interpersonal outcomes (negotiation). The indicators inter-correlated moderately in these corpora ($\rho \approx .32$–$.44$; correlations differ from the SCT-based values in Appendix B because both the text genre and the scored subset of indicators differ) but showed differential prediction, consistent with the multidimensional account of maturity developed in the main text. Effect sizes ($\rho \approx .10$–$.22$) are comparable to Big Five personality–performance associations derived from validated questionnaires. All datasets are publicly available: P4G (Wang et al., 2019), CMV (Tan et al., 2016; via ConvoKit), DeliData (Karadzhov et al., 2023; HuggingFace), CaSiNo (Chawla et al., 2021; HuggingFace), Congressional Record (Gentzkow et al., 2019; Stanford).
